# Supplementary figures and images for: An IS-mediated, RecA-dependent, bet-hedging strategy in Burkholderia thailandensis
Source: eLife. 2023 Jan 30;12:e84327. doi: 10.7554/eLife.84327 (PMC9946442; doi:10.7554/eLife.84327)

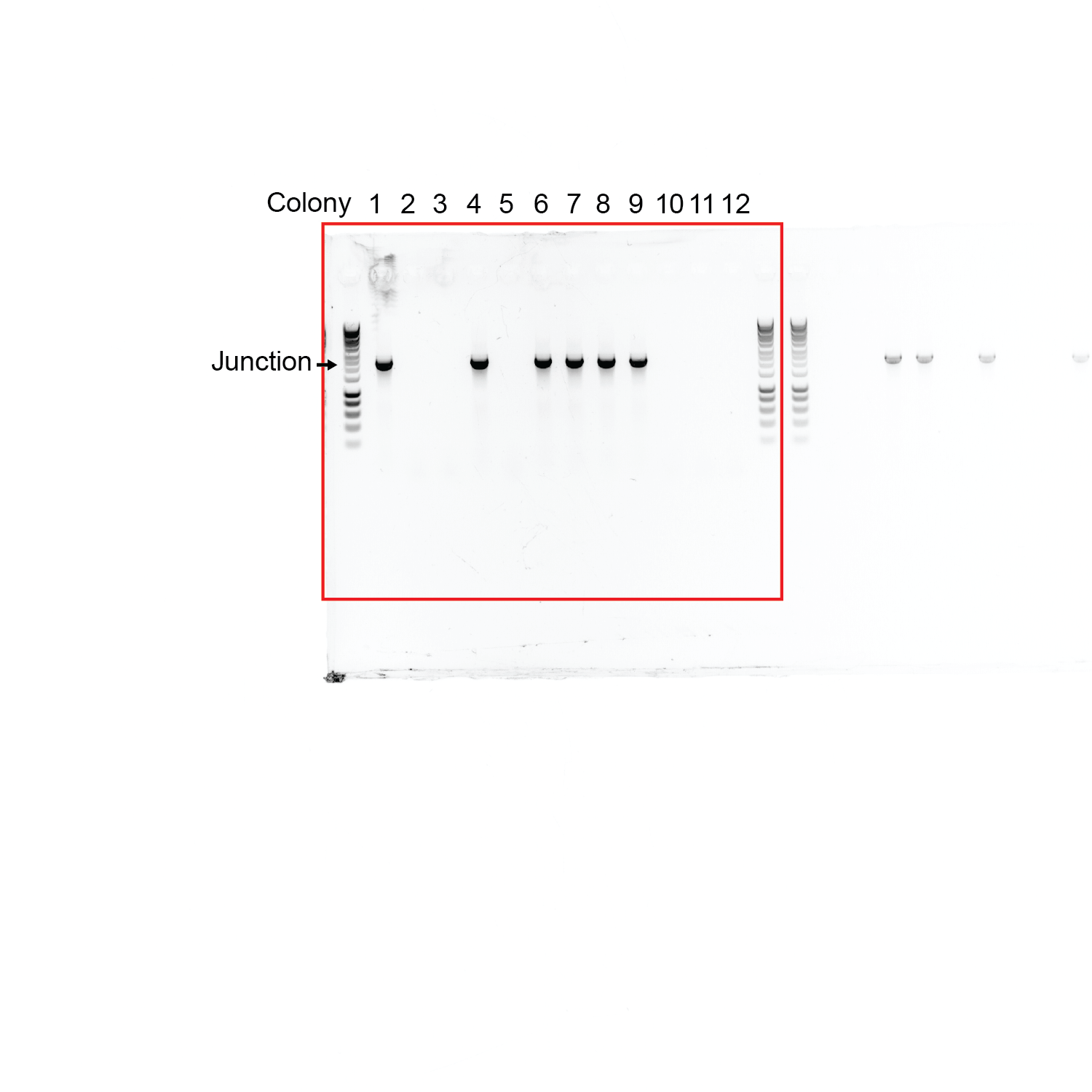

Supplement: Figure 1—source data 1. — The red box indicates the region of the gel used in the final figure. The lanes and identity of the band(s) are indicated. [file elife-84327-fig1-data1.zip › Figure 1 - Source data 1/Figure 1 - Source data 1.png]

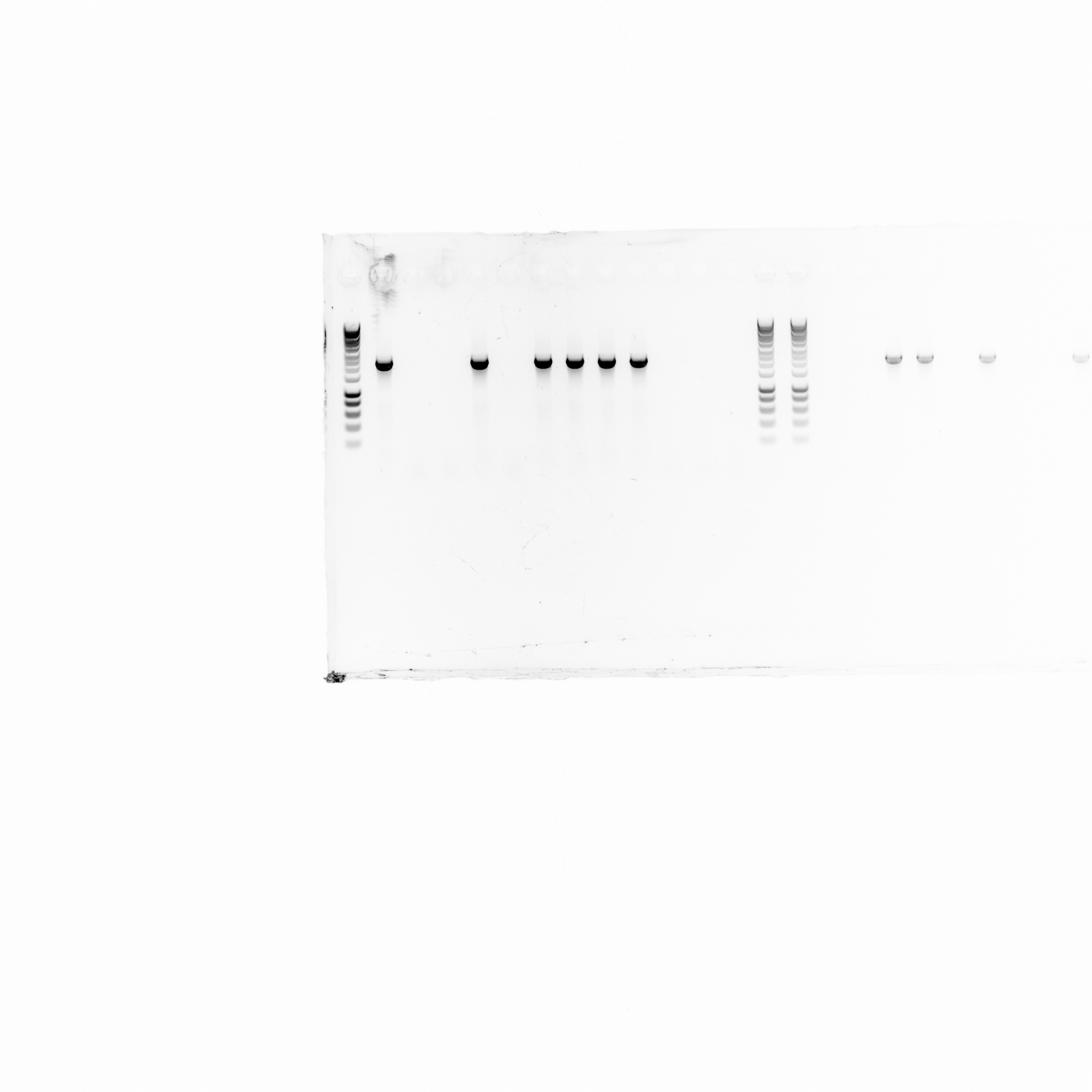

Supplement: Figure 1—source data 1. — The red box indicates the region of the gel used in the final figure. The lanes and identity of the band(s) are indicated. [file elife-84327-fig1-data1.zip › Figure 1 - Source data 1/Figure 1- Source data 1 unedited.tif]

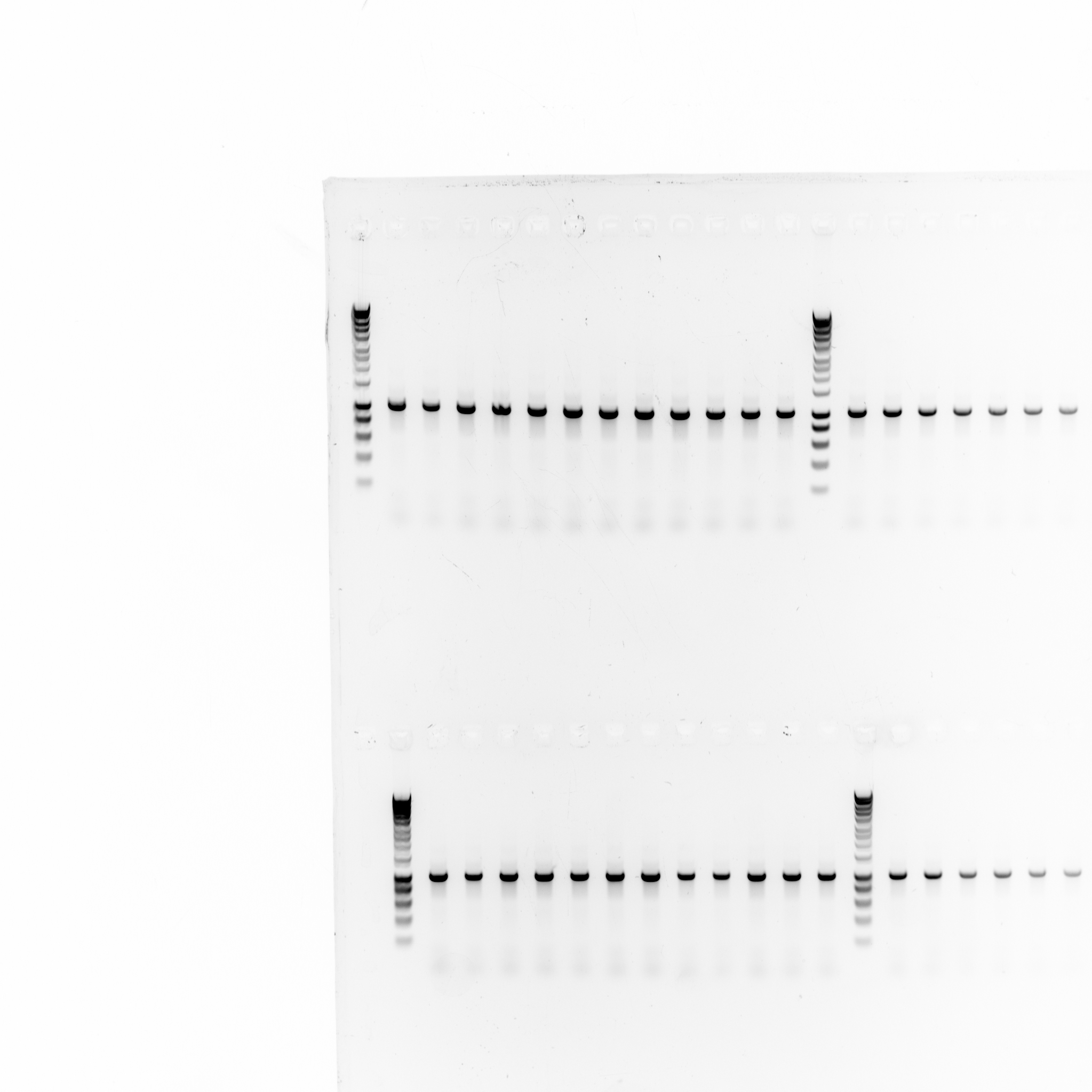

Supplement: Figure 1—source data 2. — The red box indicates the region of the gel used in the final figure. The lanes and identity of the band(s) are indicated. [file elife-84327-fig1-data2.zip › Figure 1 - Source data 2/Figure 1 - Source data 2 unedited.tif]

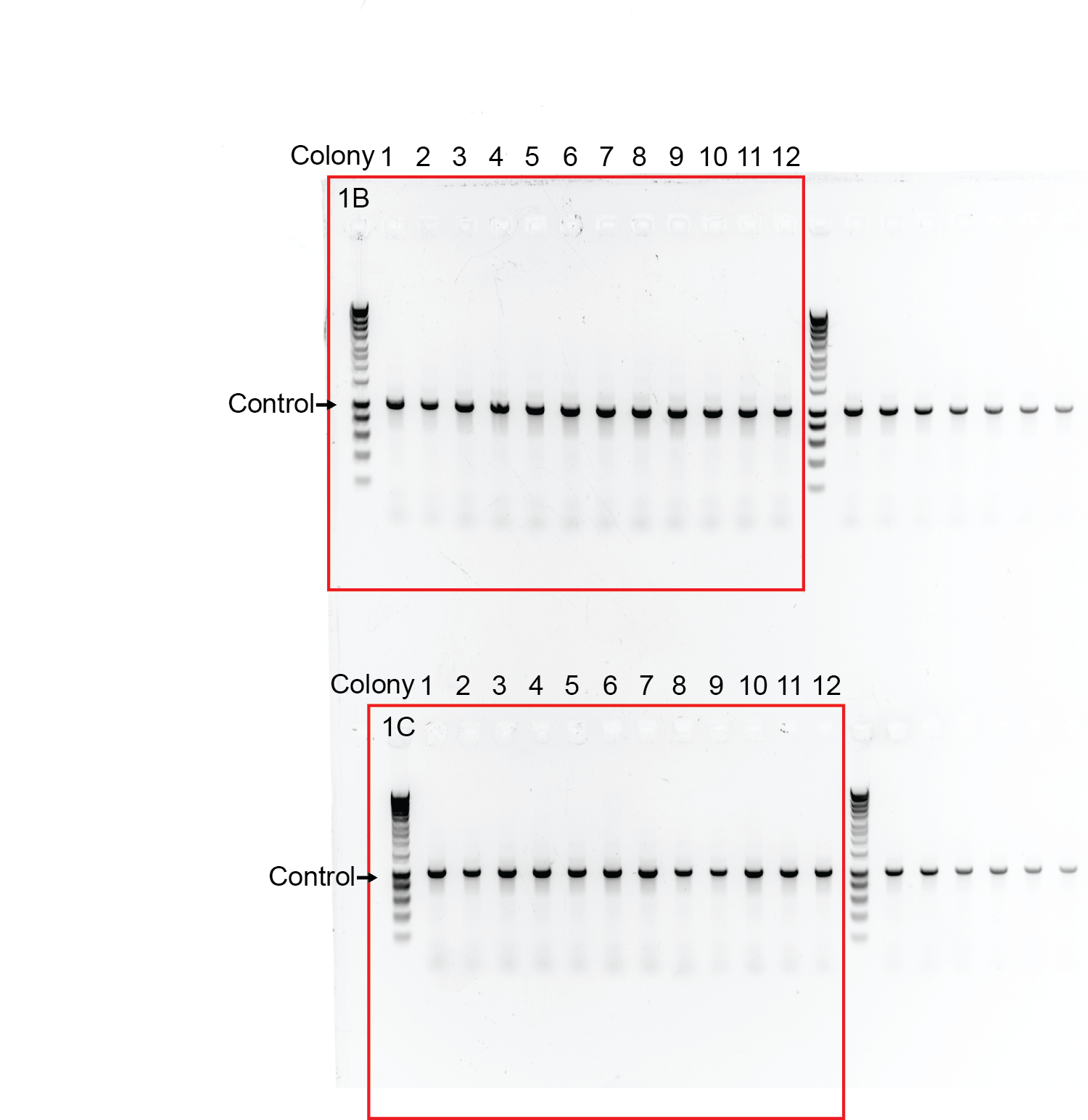

Supplement: Figure 1—source data 2. — The red box indicates the region of the gel used in the final figure. The lanes and identity of the band(s) are indicated. [file elife-84327-fig1-data2.zip › Figure 1 - Source data 2/Figure 1 - Source data 2.png]

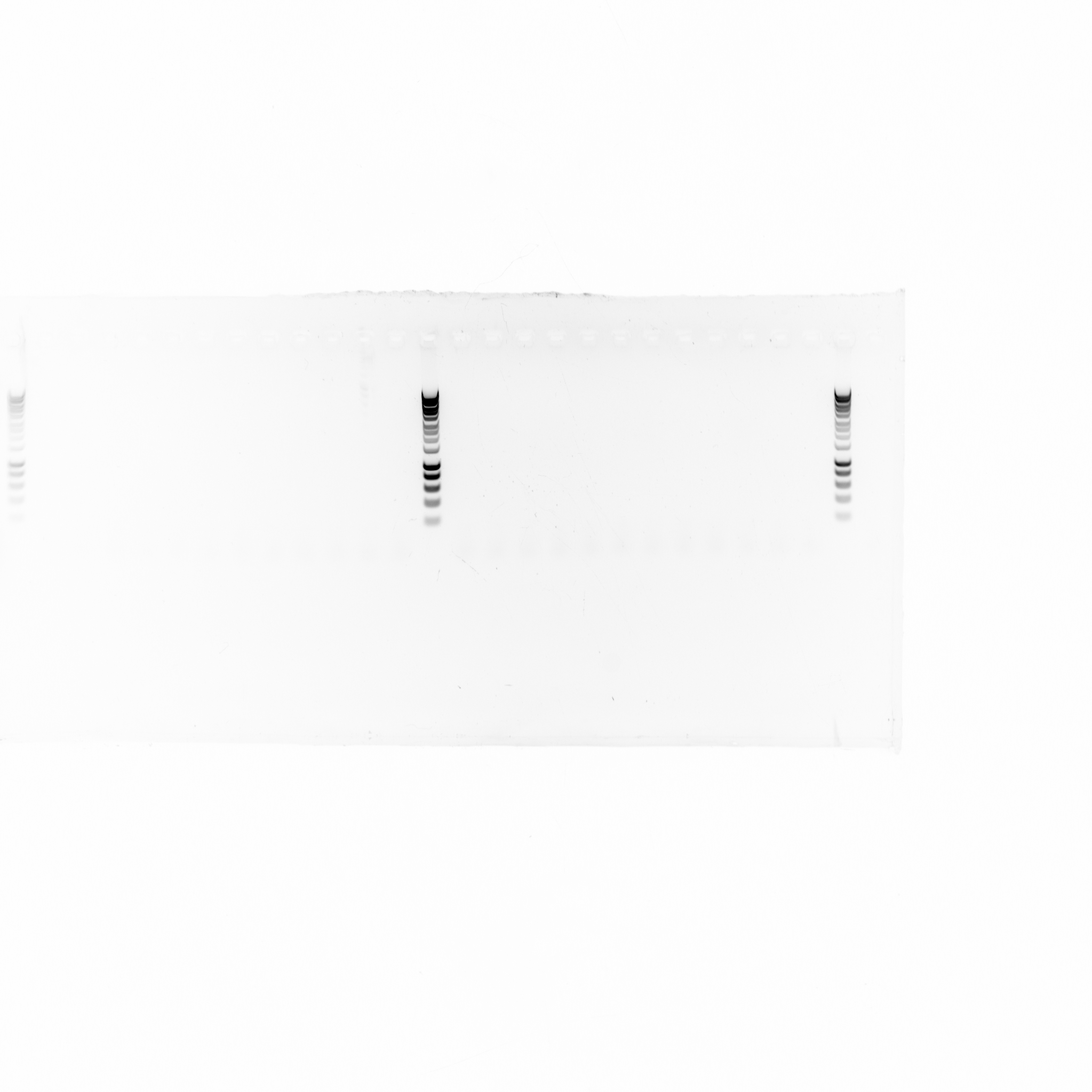

Supplement: Figure 1—source data 3. — The red box indicates the region of the gel used in the final figure. The lanes and identity of the band(s) are indicated. [file elife-84327-fig1-data3.zip › Figure 1 - Source data 3/Figure 1 - Source data 3 unedited.tif]

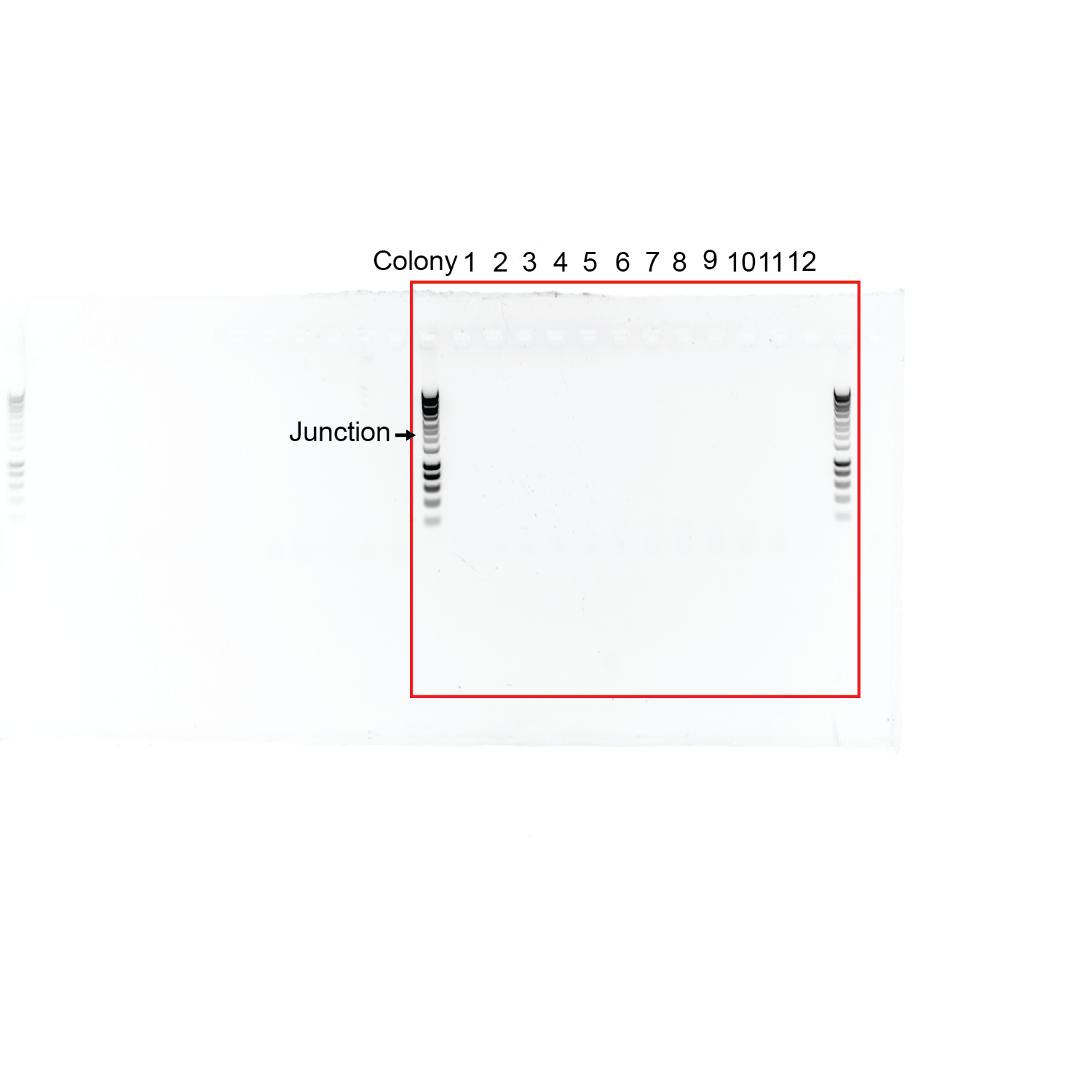

Supplement: Figure 1—source data 3. — The red box indicates the region of the gel used in the final figure. The lanes and identity of the band(s) are indicated. [file elife-84327-fig1-data3.zip › Figure 1 - Source data 3/Figure 1 - Source data 3.png]

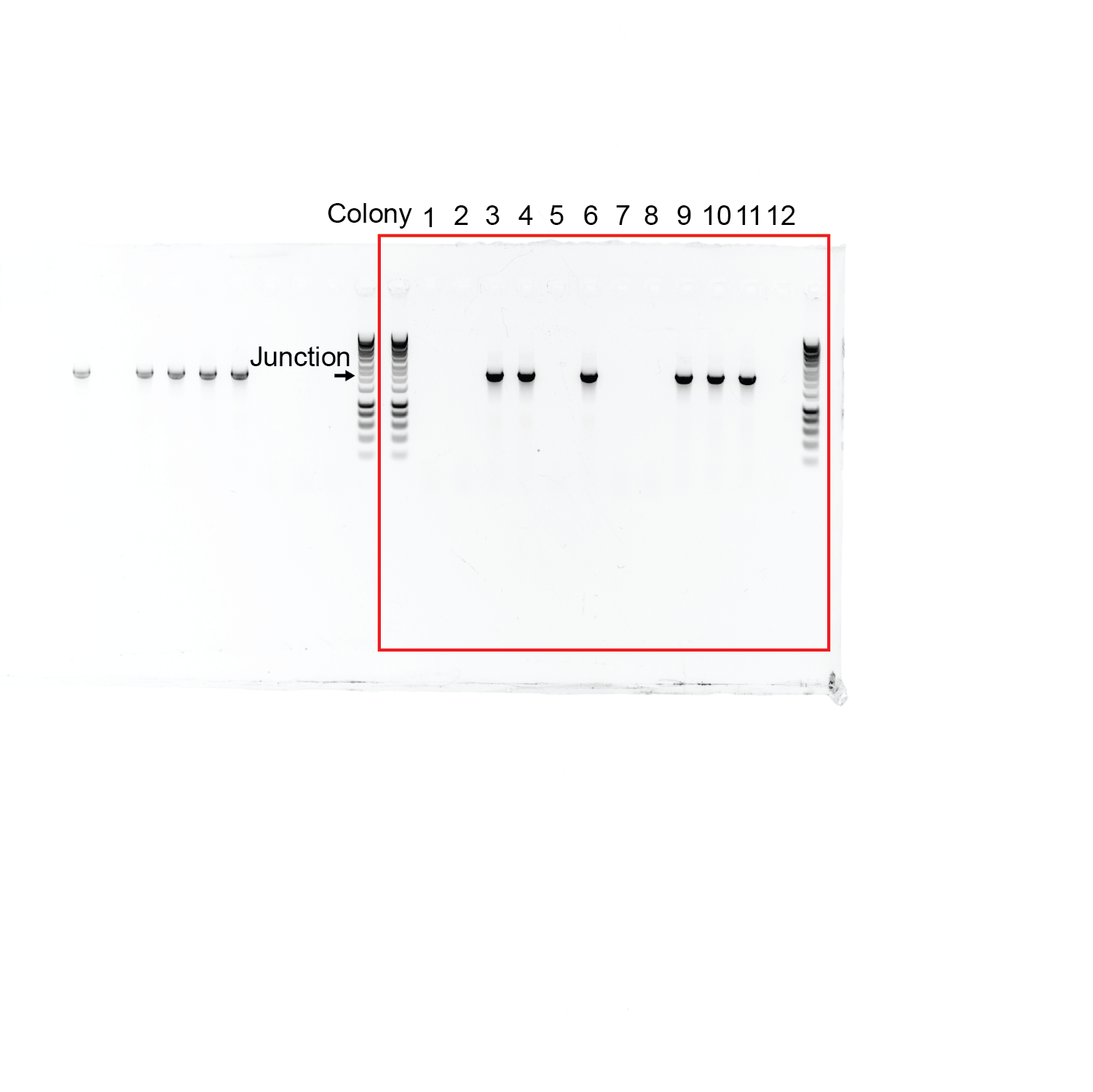

Supplement: Figure 1—source data 4. — The red box indicates the region of the gel used in the final figure. The lanes and identity of the band(s) are indicated. [file elife-84327-fig1-data4.zip › Figure 1 - Source data 4/Figure 1 - Source data 4.png]

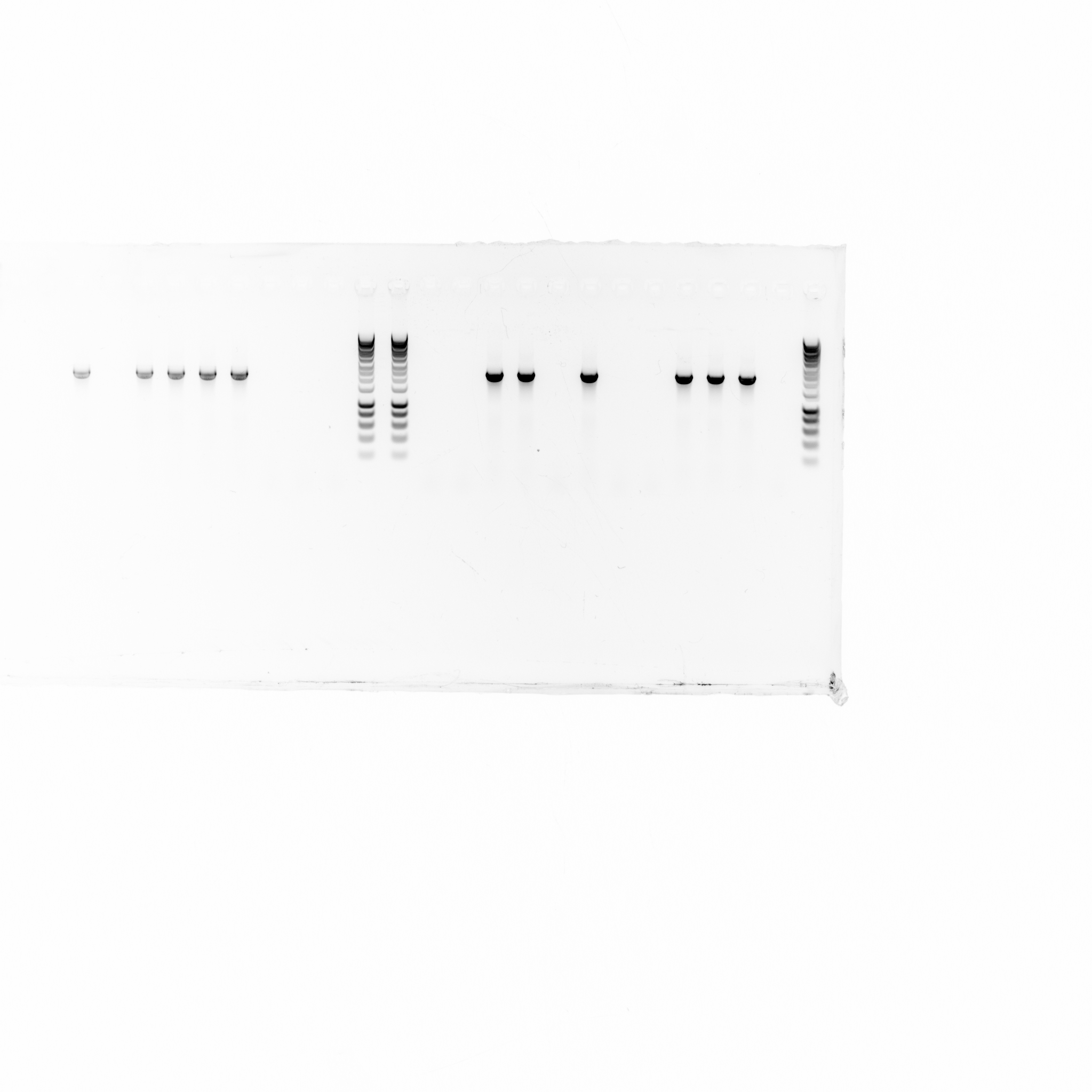

Supplement: Figure 1—source data 4. — The red box indicates the region of the gel used in the final figure. The lanes and identity of the band(s) are indicated. [file elife-84327-fig1-data4.zip › Figure 1 - Source data 4/Figure 1 - Source figure 4 unedited.tif]

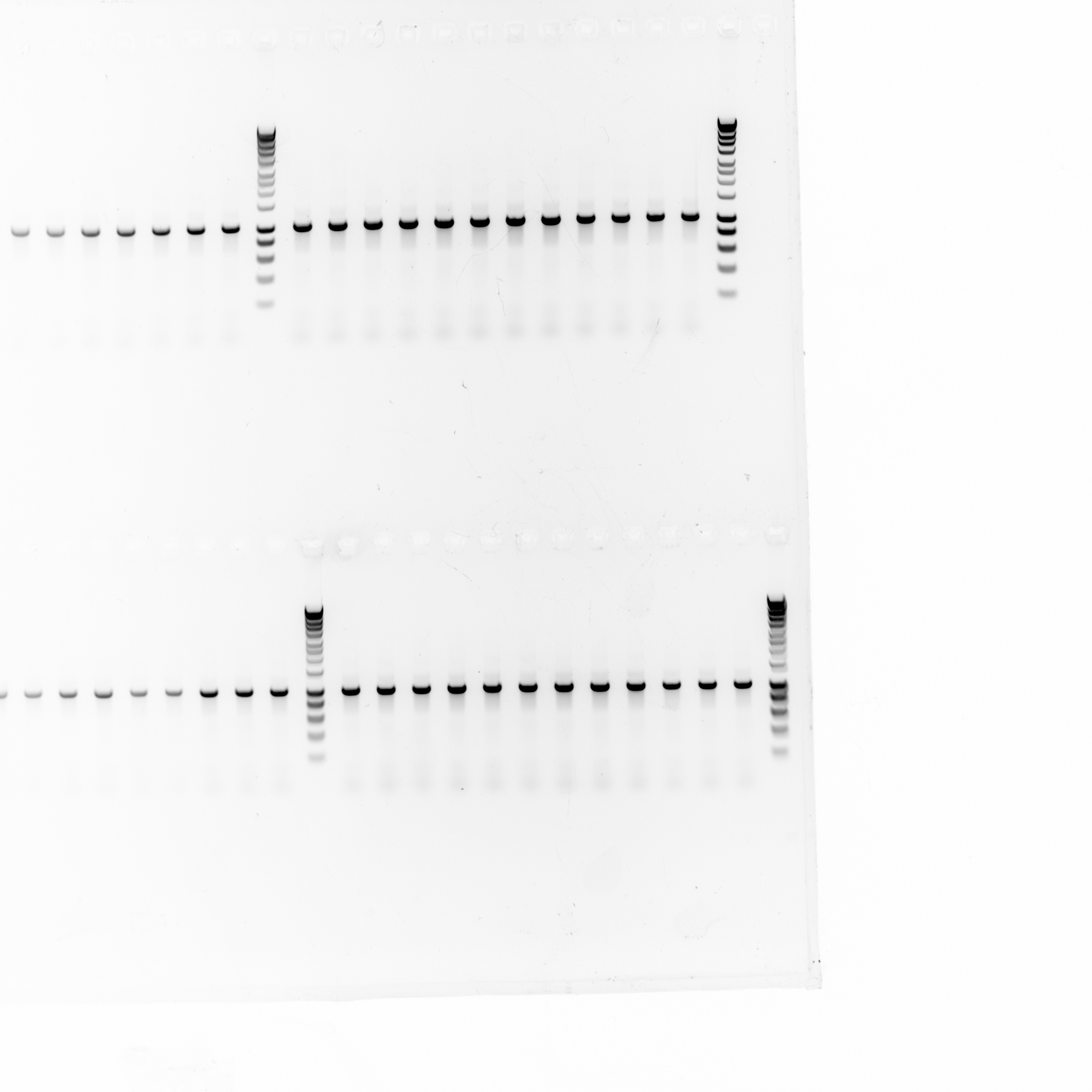

Supplement: Figure 1—source data 5. — The red box indicates the region of the gel used in the final figure. The lanes and identity of the band(s) are indicated. [file elife-84327-fig1-data5.zip › Figure 1 - Source data 5/Figure 1 - Source data 5 unedited.tif]

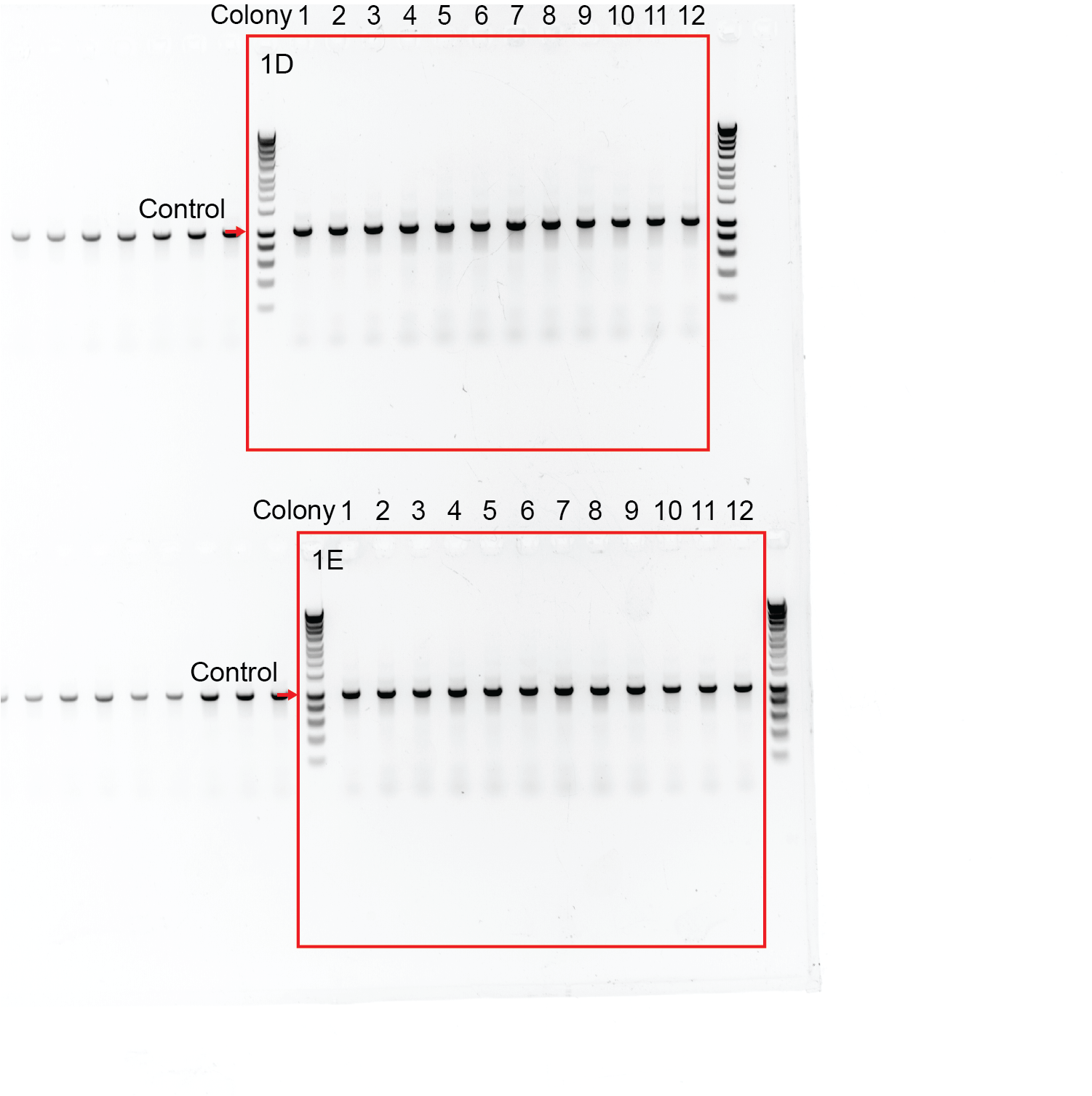

Supplement: Figure 1—source data 5. — The red box indicates the region of the gel used in the final figure. The lanes and identity of the band(s) are indicated. [file elife-84327-fig1-data5.zip › Figure 1 - Source data 5/Figure 1 - Source data 5.png]

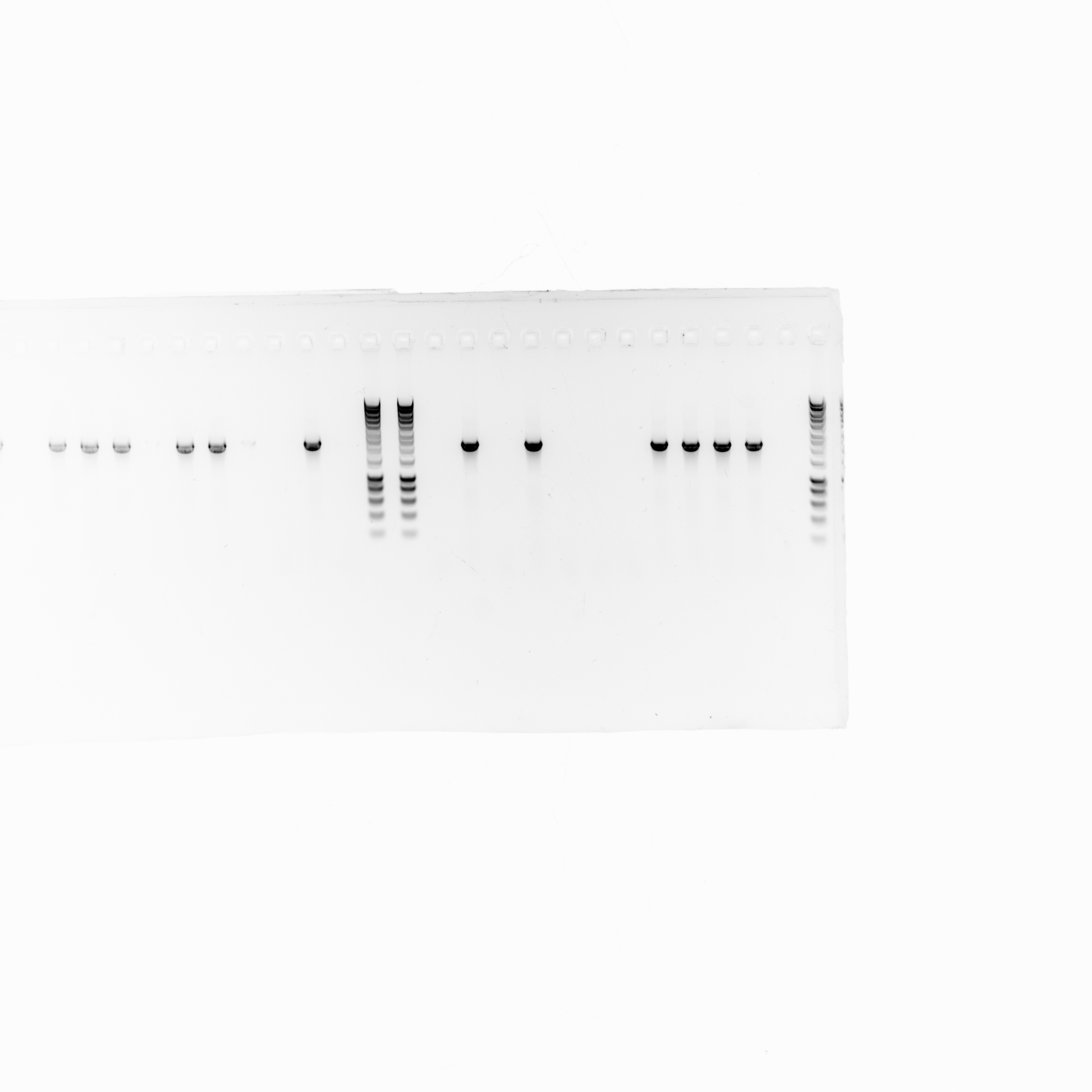

Supplement: Figure 1—source data 6. — The red box indicates the region of the gel used in the final figure. The lanes and identity of the band(s) are indicated. [file elife-84327-fig1-data6.zip › Figure 1 - Source data 6/Figure 1 - source data 6 unedited.tif]

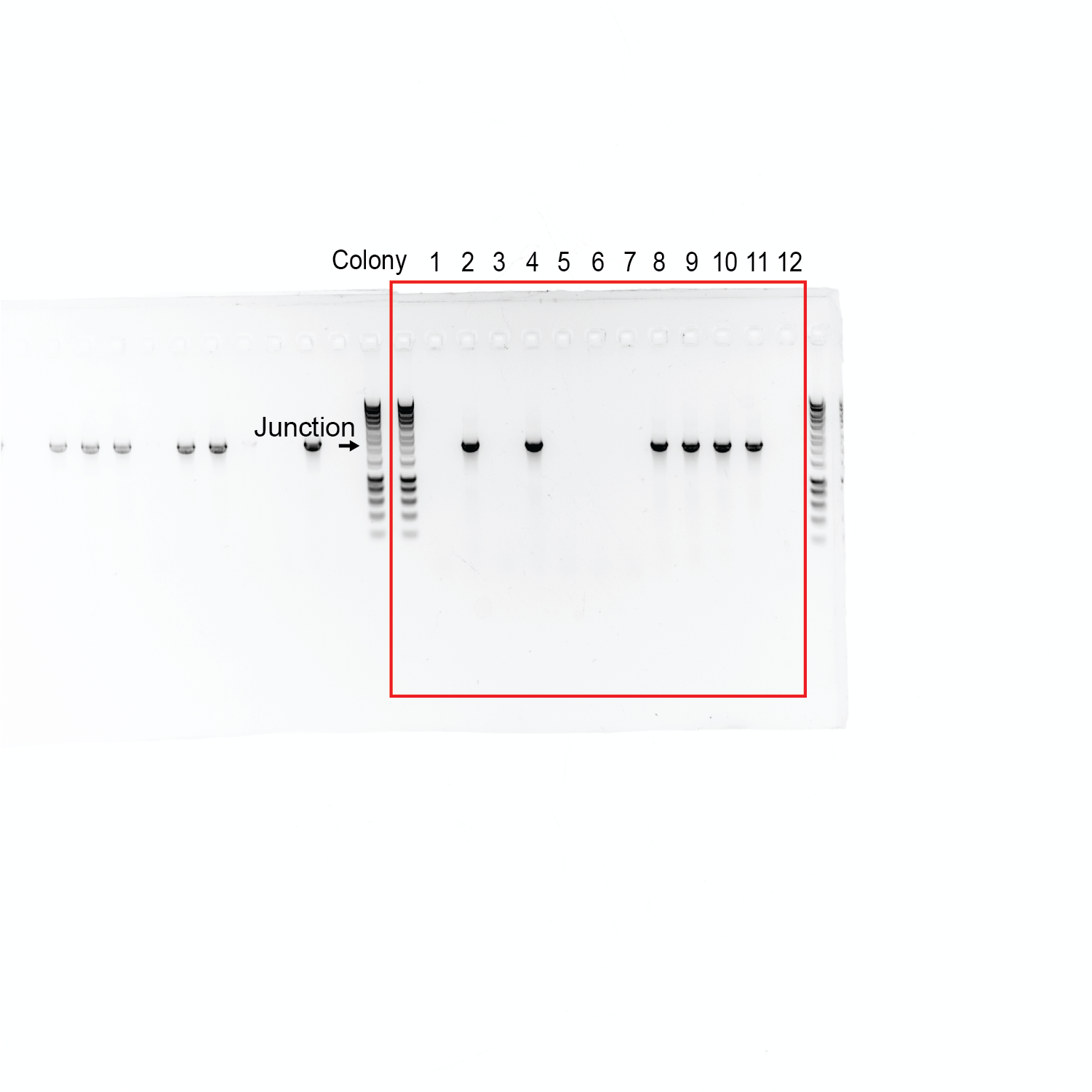

Supplement: Figure 1—source data 6. — The red box indicates the region of the gel used in the final figure. The lanes and identity of the band(s) are indicated. [file elife-84327-fig1-data6.zip › Figure 1 - Source data 6/Figure 1 - Source data 6.png]

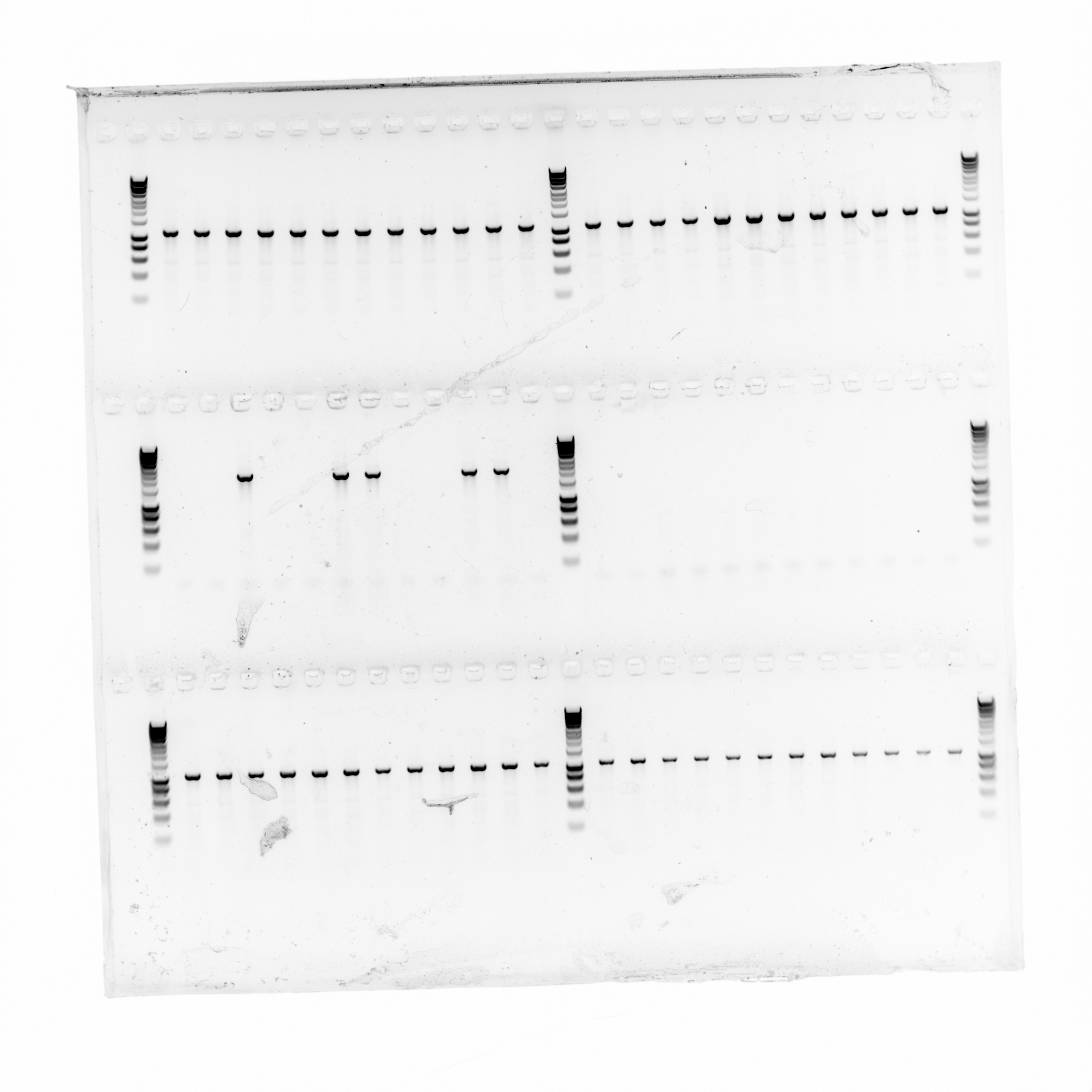

Supplement: Figure 1—source data 7. — The red box indicates the region of the gel used in the final figure. The lanes and identity of the band(s) are indicated. [file elife-84327-fig1-data7.zip › Figure 1 - Source data 7/Figure 1 - Source data 7 unedited.tif]

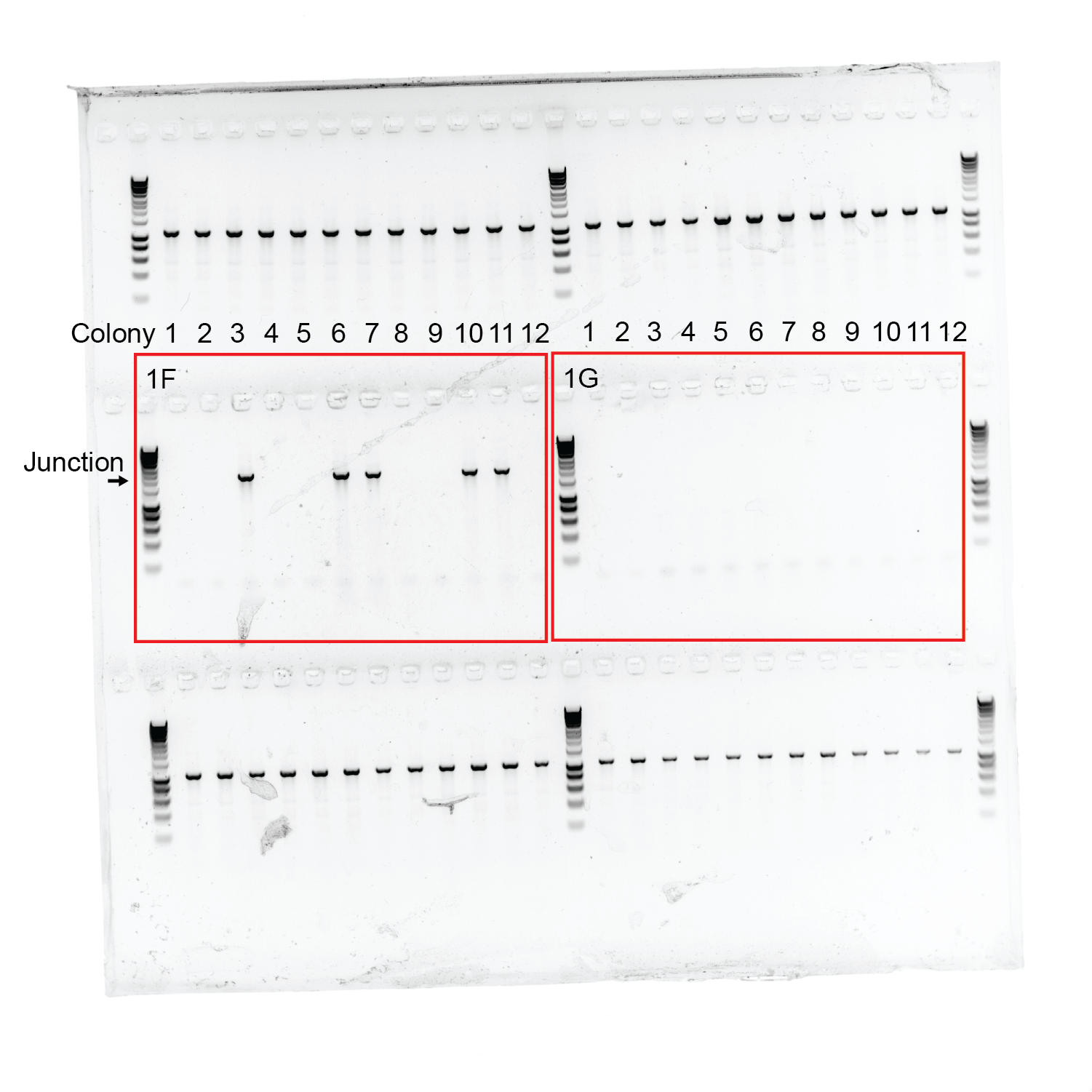

Supplement: Figure 1—source data 7. — The red box indicates the region of the gel used in the final figure. The lanes and identity of the band(s) are indicated. [file elife-84327-fig1-data7.zip › Figure 1 - Source data 7/Figure 1 - Source data 7.png]

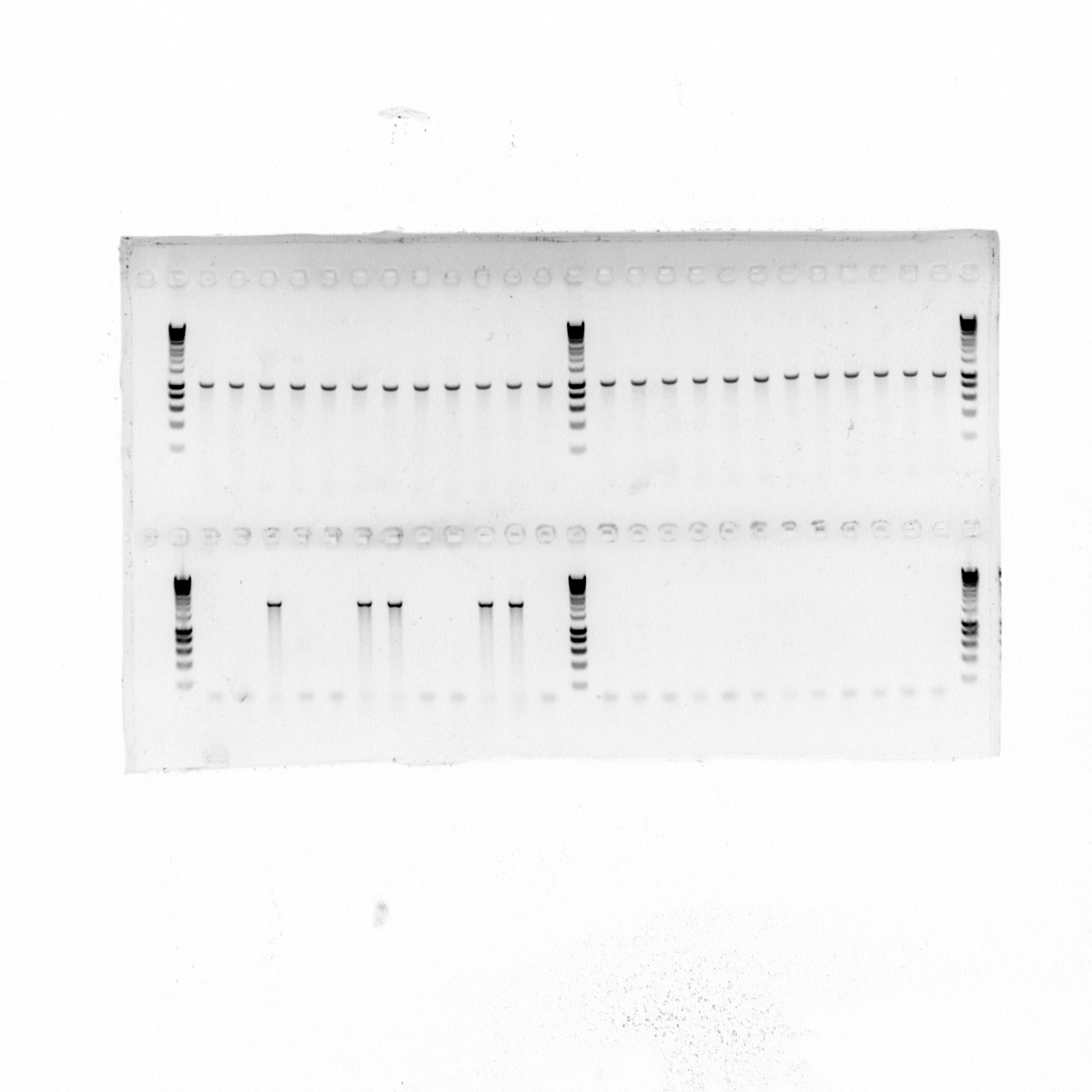

Supplement: Figure 1—source data 8. — The red box indicates the region of the gel used in the final figure. The lanes and identity of the band(s) are indicated. [file elife-84327-fig1-data8.zip › Figure 1 - Source data 8/Figure 1 - Source data 8 unedited.tif]

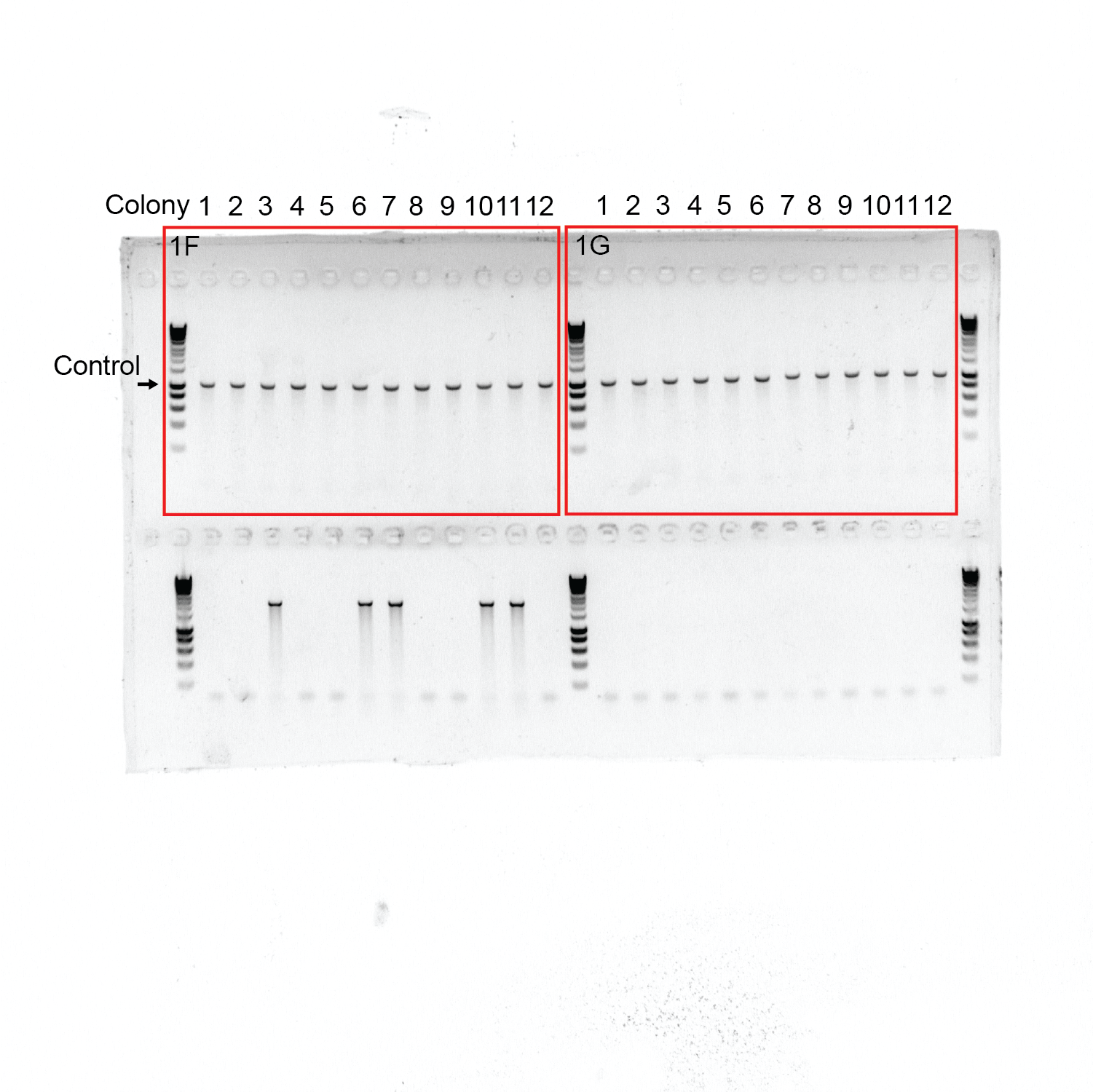

Supplement: Figure 1—source data 8. — The red box indicates the region of the gel used in the final figure. The lanes and identity of the band(s) are indicated. [file elife-84327-fig1-data8.zip › Figure 1 - Source data 8/Figure 1 - Source data 8.png]

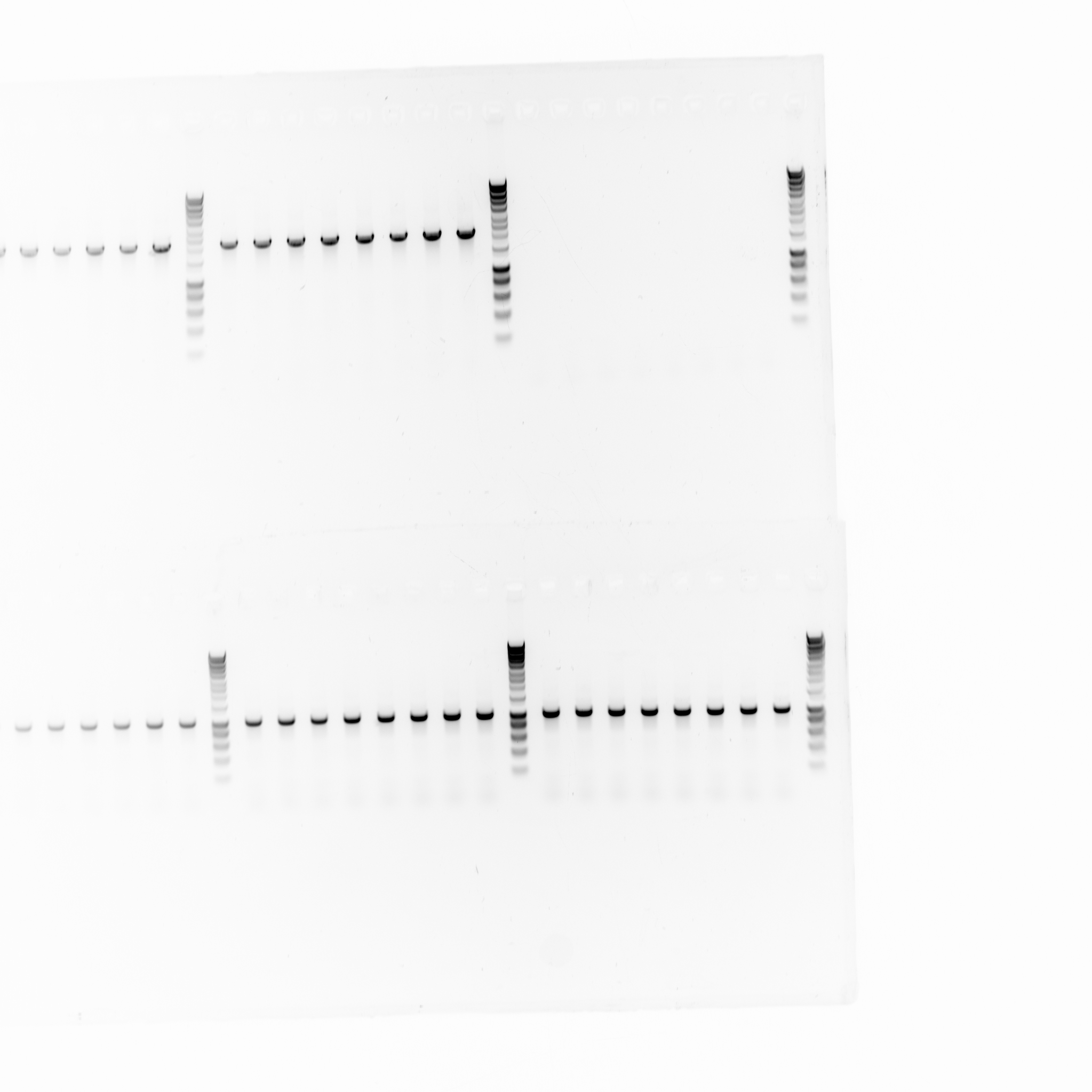

Supplement: Figure 2—source data 1. — The red box indicates the region of the gel used in the final figure. The lanes and identity of the band(s) are indicated. [file elife-84327-fig2-data1.zip › Figure 2 - Source data 1/Figure 2 - Source data 1 unedited.tif]

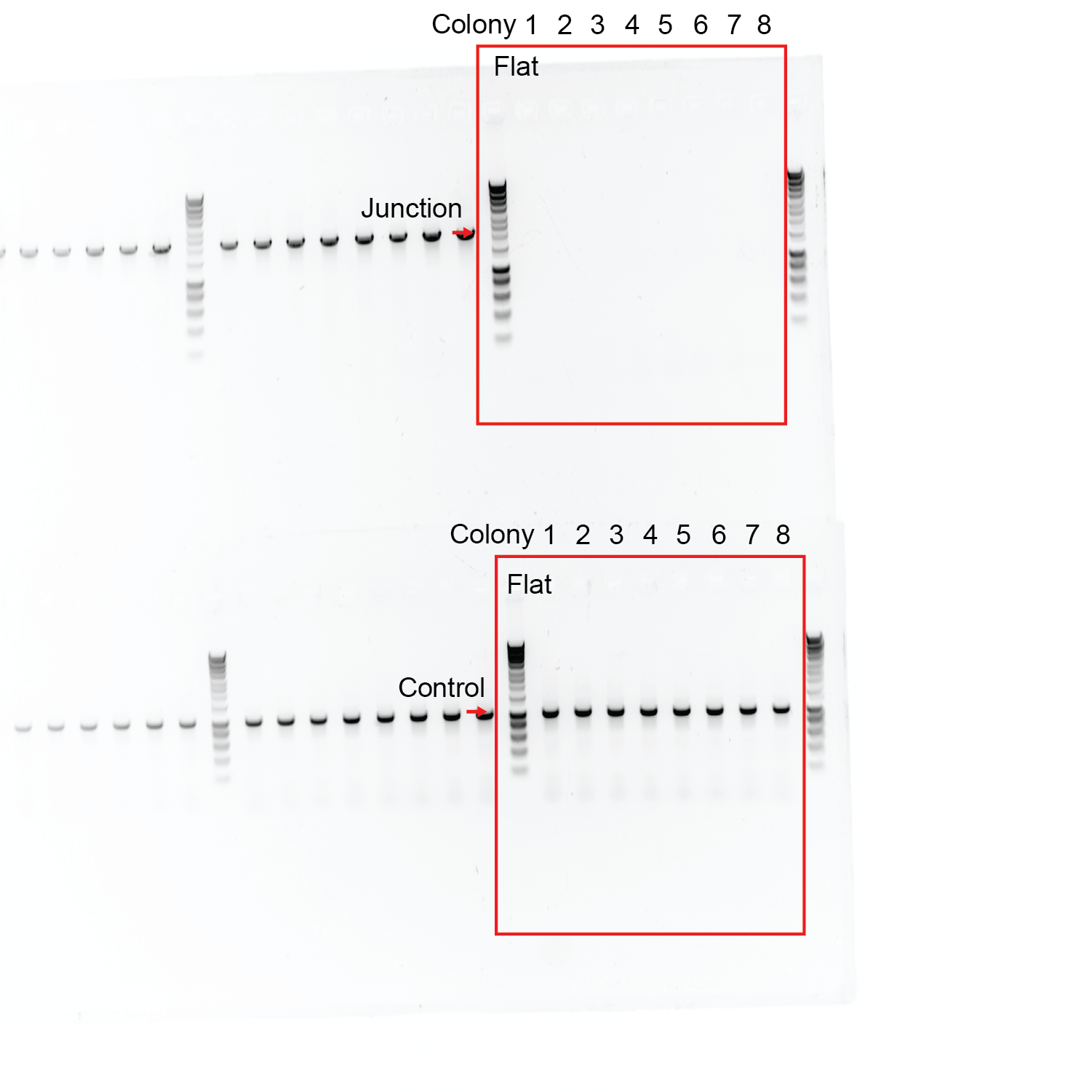

Supplement: Figure 2—source data 1. — The red box indicates the region of the gel used in the final figure. The lanes and identity of the band(s) are indicated. [file elife-84327-fig2-data1.zip › Figure 2 - Source data 1/Figure 2 - Source data 1.png]

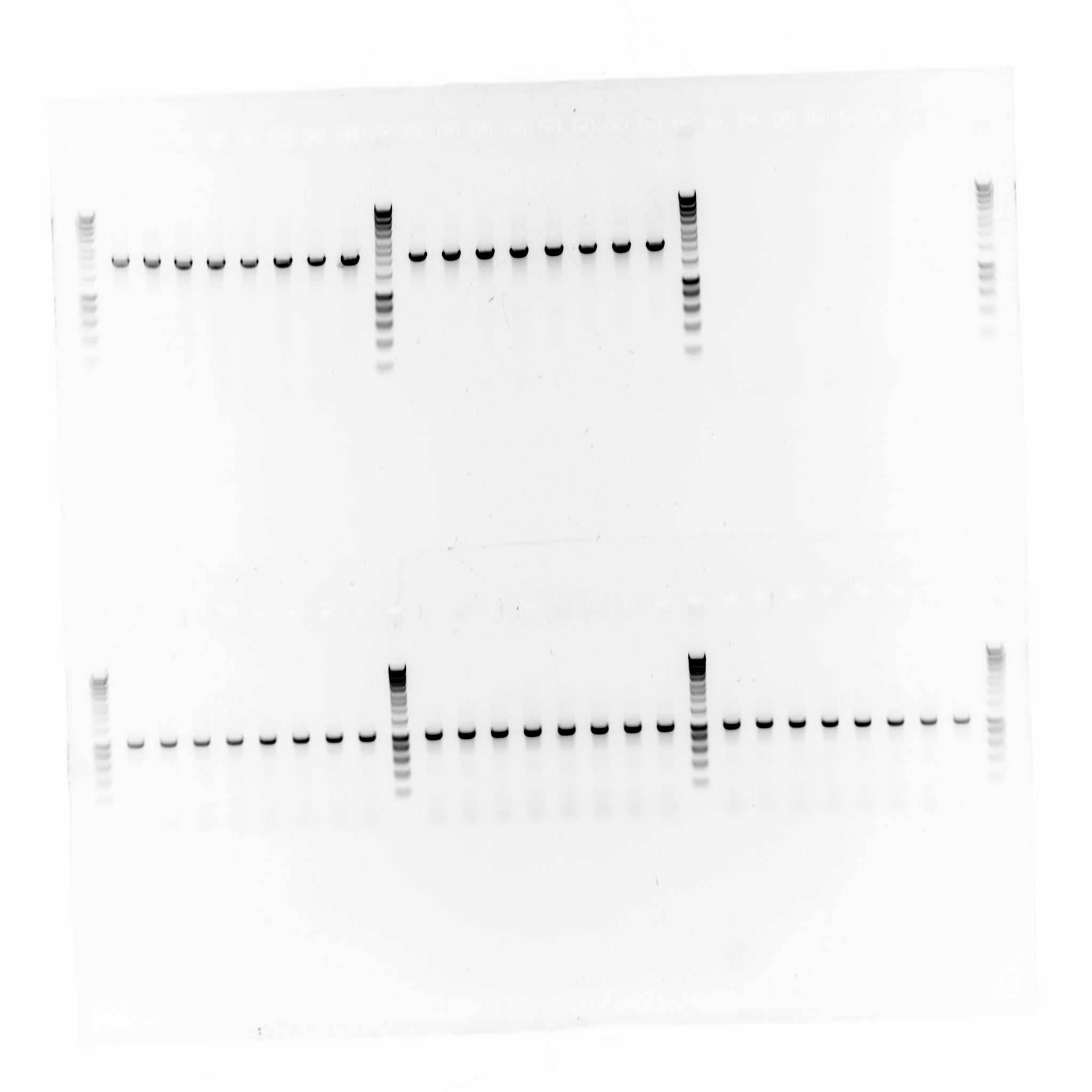

Supplement: Figure 2—source data 2. — The red box indicates the region of the gel used in the final figure. The lanes and identity of the band(s) are indicated. [file elife-84327-fig2-data2.zip › Figure 2 - Source data 2/Figure 2 - Source data 2 Unedited.tif]

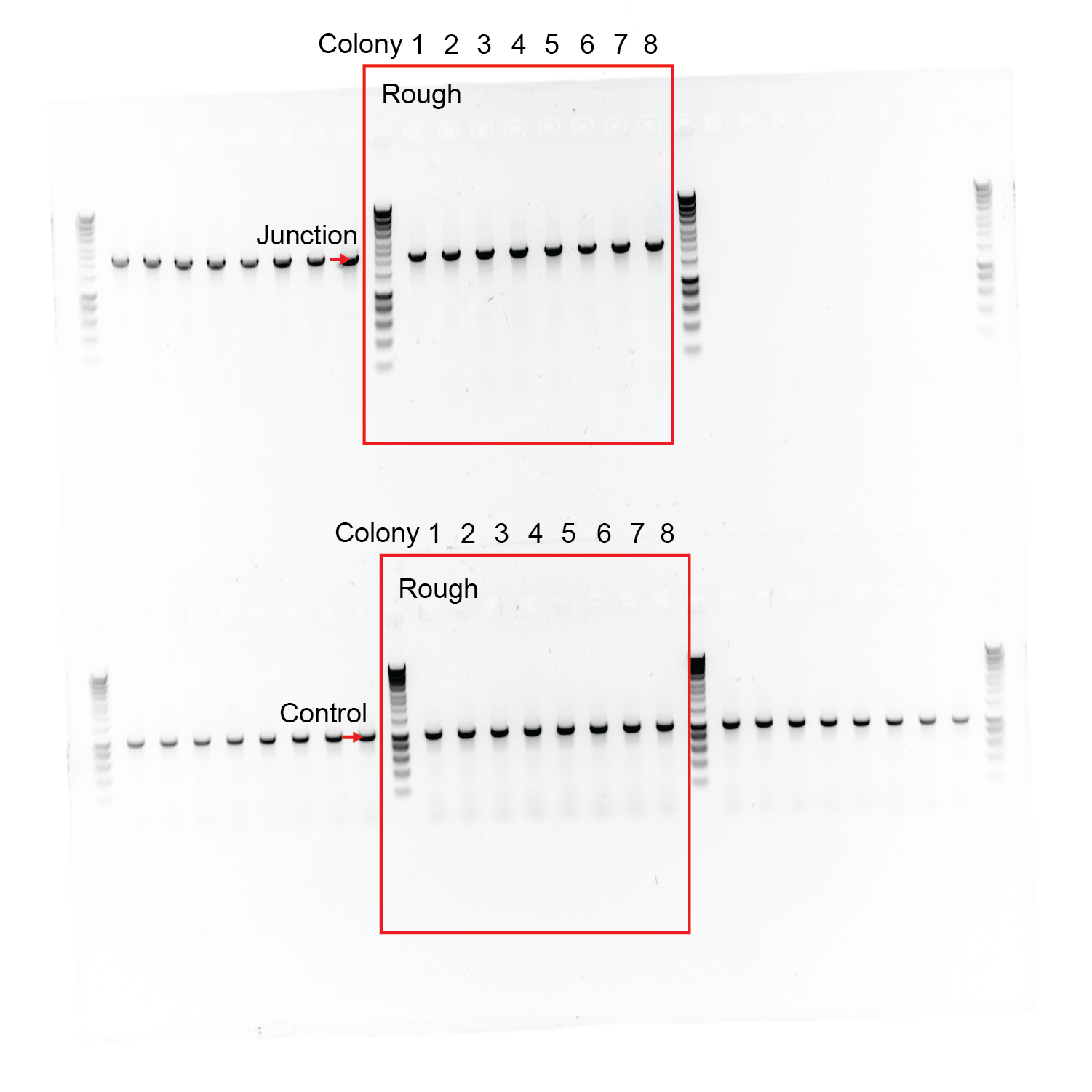

Supplement: Figure 2—source data 2. — The red box indicates the region of the gel used in the final figure. The lanes and identity of the band(s) are indicated. [file elife-84327-fig2-data2.zip › Figure 2 - Source data 2/Figure 2 - Source data 2.png]

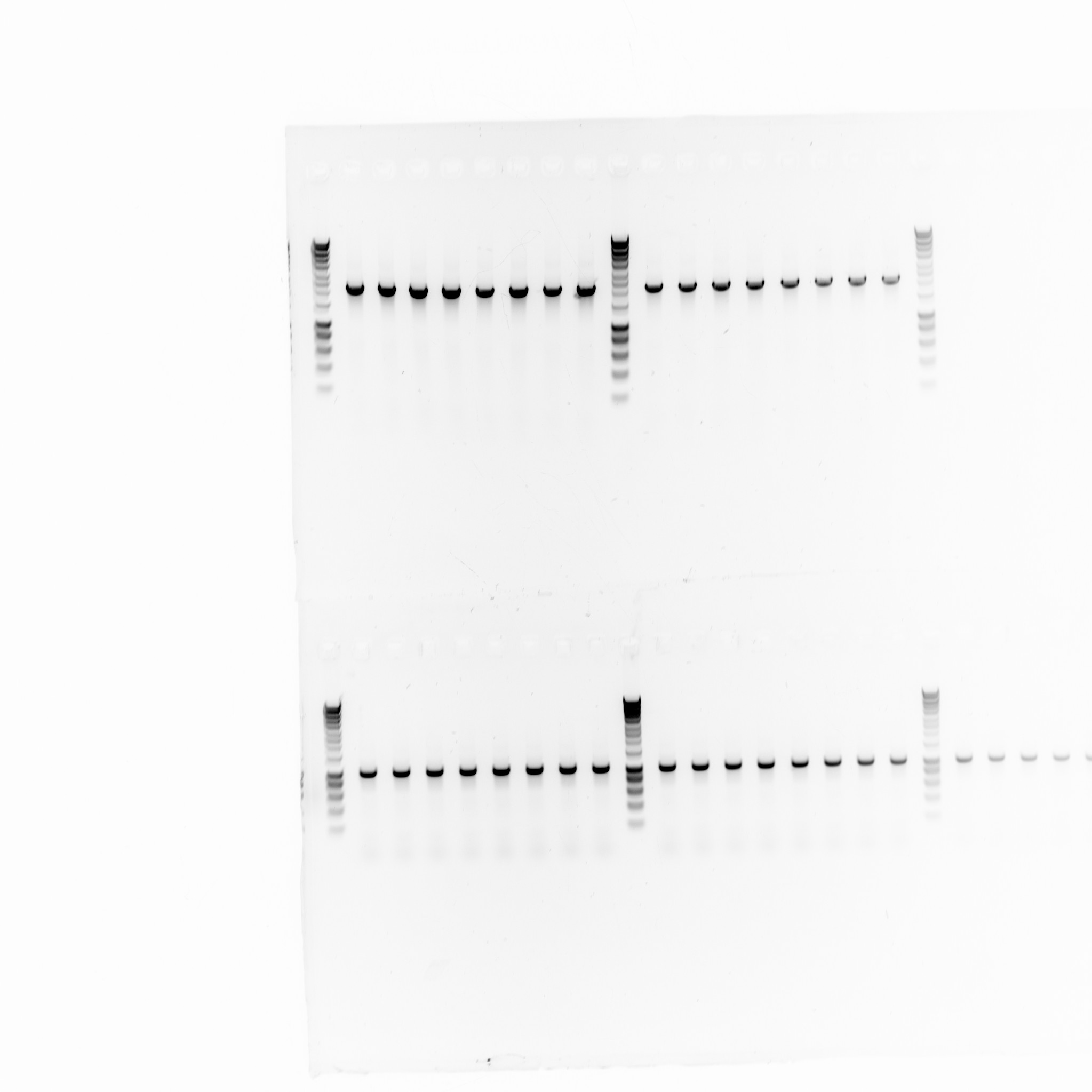

Supplement: Figure 2—source data 3. — The red box indicates the region of the gel used in the final figure. The lanes and identity of the band(s) are indicated. [file elife-84327-fig2-data3.zip › Figure 2 - Source data 3/Figure 2 - Source data 3 unedited.tif]

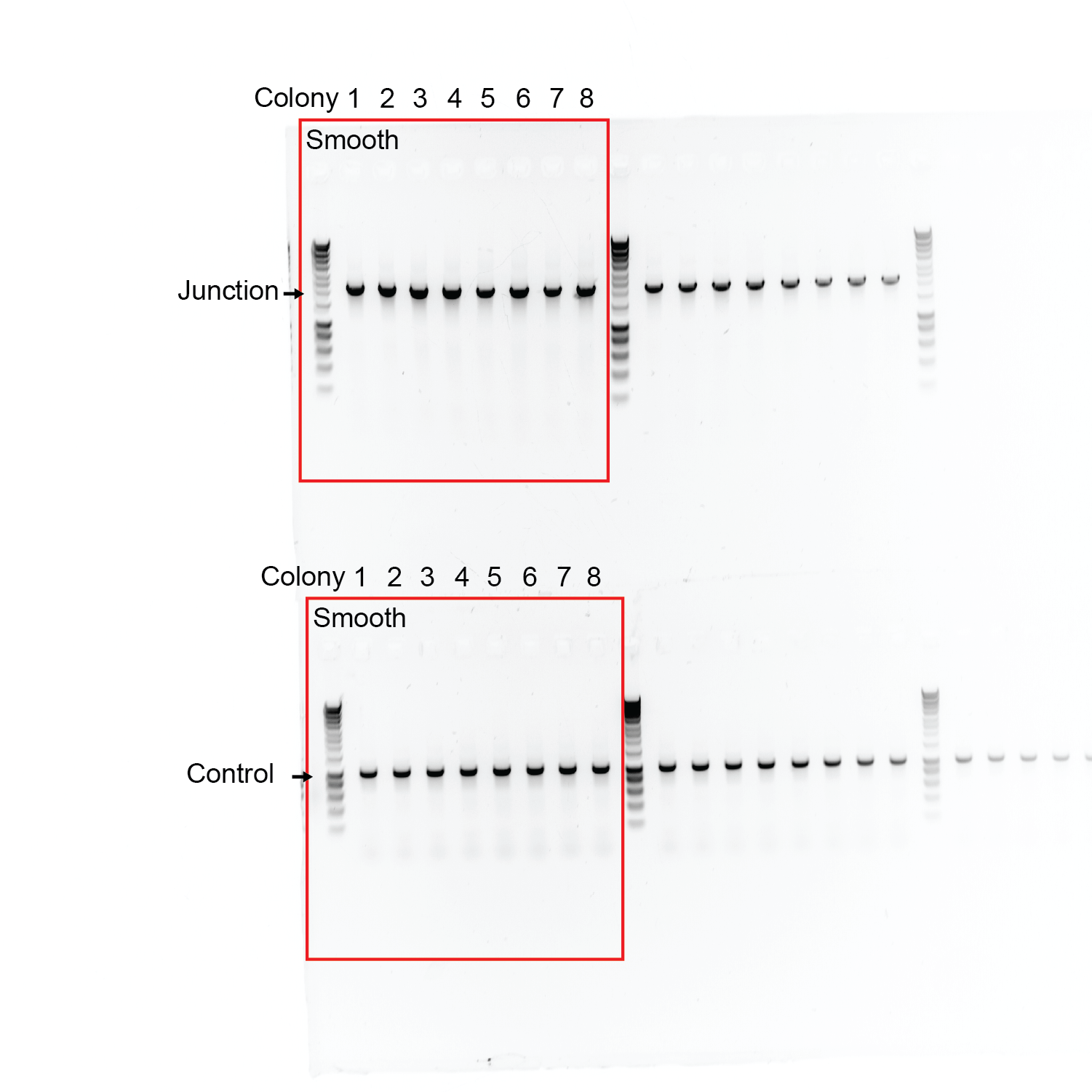

Supplement: Figure 2—source data 3. — The red box indicates the region of the gel used in the final figure. The lanes and identity of the band(s) are indicated. [file elife-84327-fig2-data3.zip › Figure 2 - Source data 3/Figure 2 - Source data 3.png]

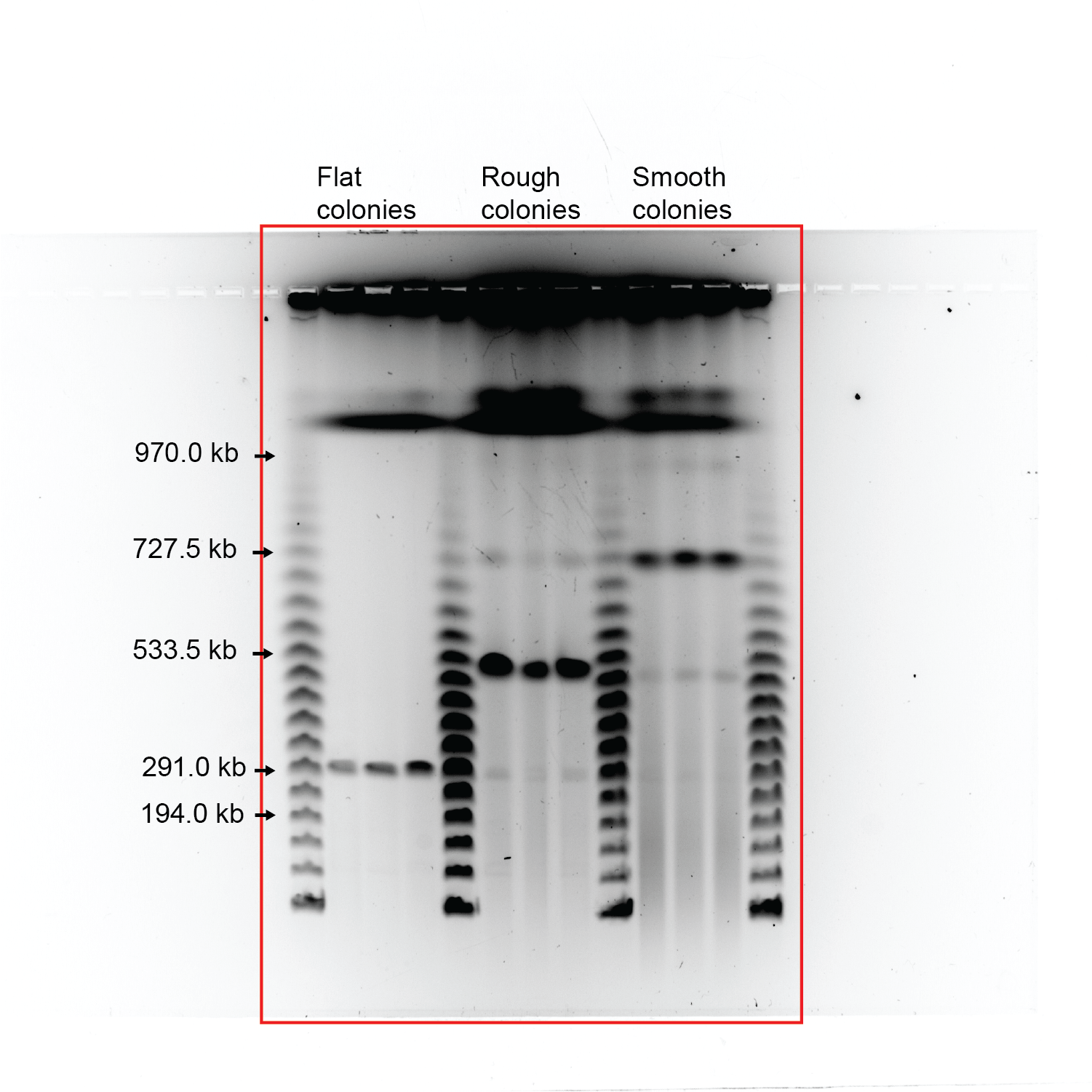

Supplement: Figure 3—source data 1. — The red box indicates the region of the gel used in the final figure. The lanes and identity of the band(s) are indicated. [file elife-84327-fig3-data1.zip › Figure 3 - Source data 1/Figure 3 - Source data 1.png]

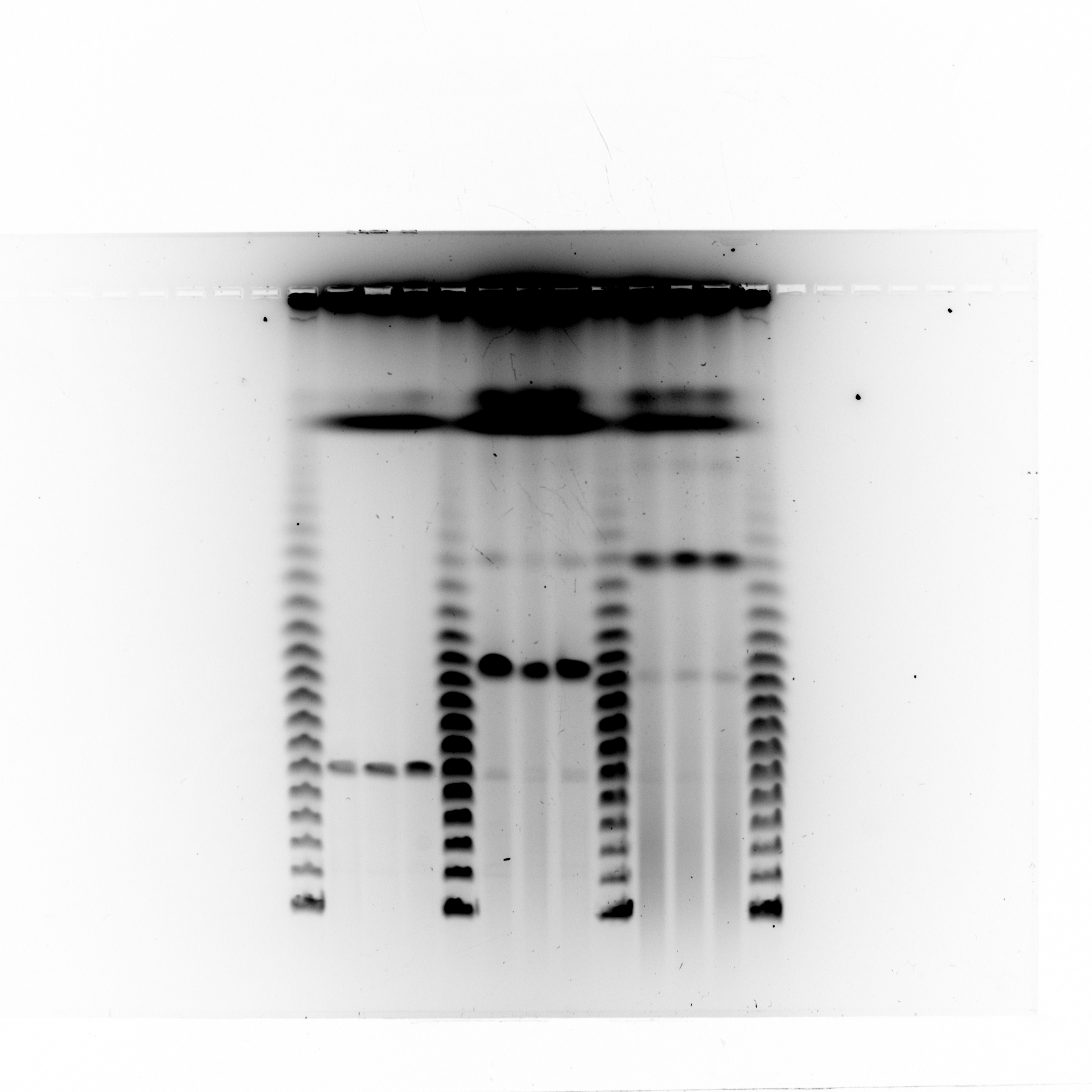

Supplement: Figure 3—source data 1. — The red box indicates the region of the gel used in the final figure. The lanes and identity of the band(s) are indicated. [file elife-84327-fig3-data1.zip › Figure 3 - Source data 1/Figure 3 - Source data 1.tif]

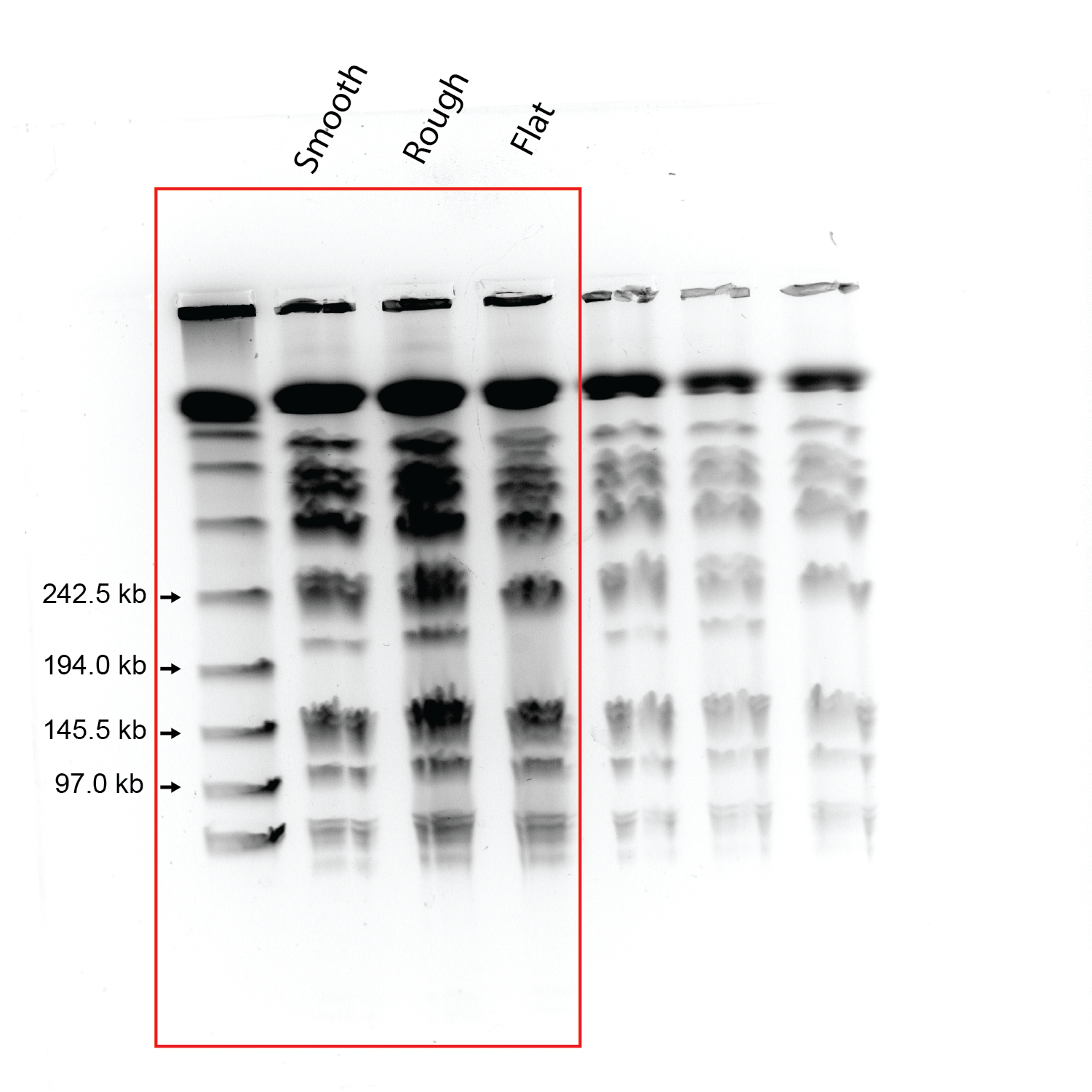

Supplement: Figure 3—figure supplement 1—source data 1. — The red box indicates the region of the gel used in the final figure. The lanes and identity of the band(s) are indicated. [file elife-84327-fig3-figsupp1-data1.zip › Figure 3 - Source data 2/Figure 3 - Source data 2.png]

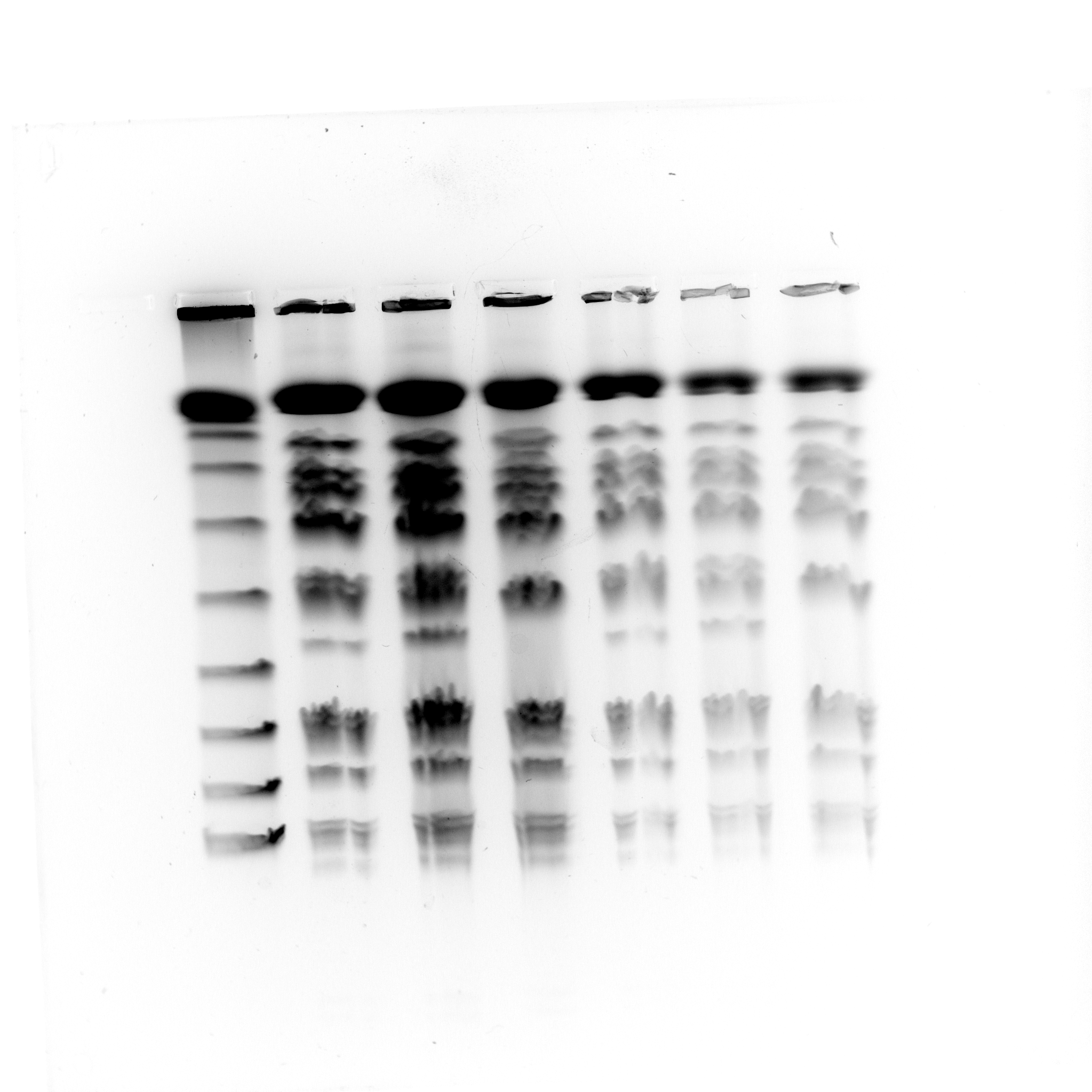

Supplement: Figure 3—figure supplement 1—source data 1. — The red box indicates the region of the gel used in the final figure. The lanes and identity of the band(s) are indicated. [file elife-84327-fig3-figsupp1-data1.zip › Figure 3 - Source data 2/Figure 3 - Source data 2.tif]

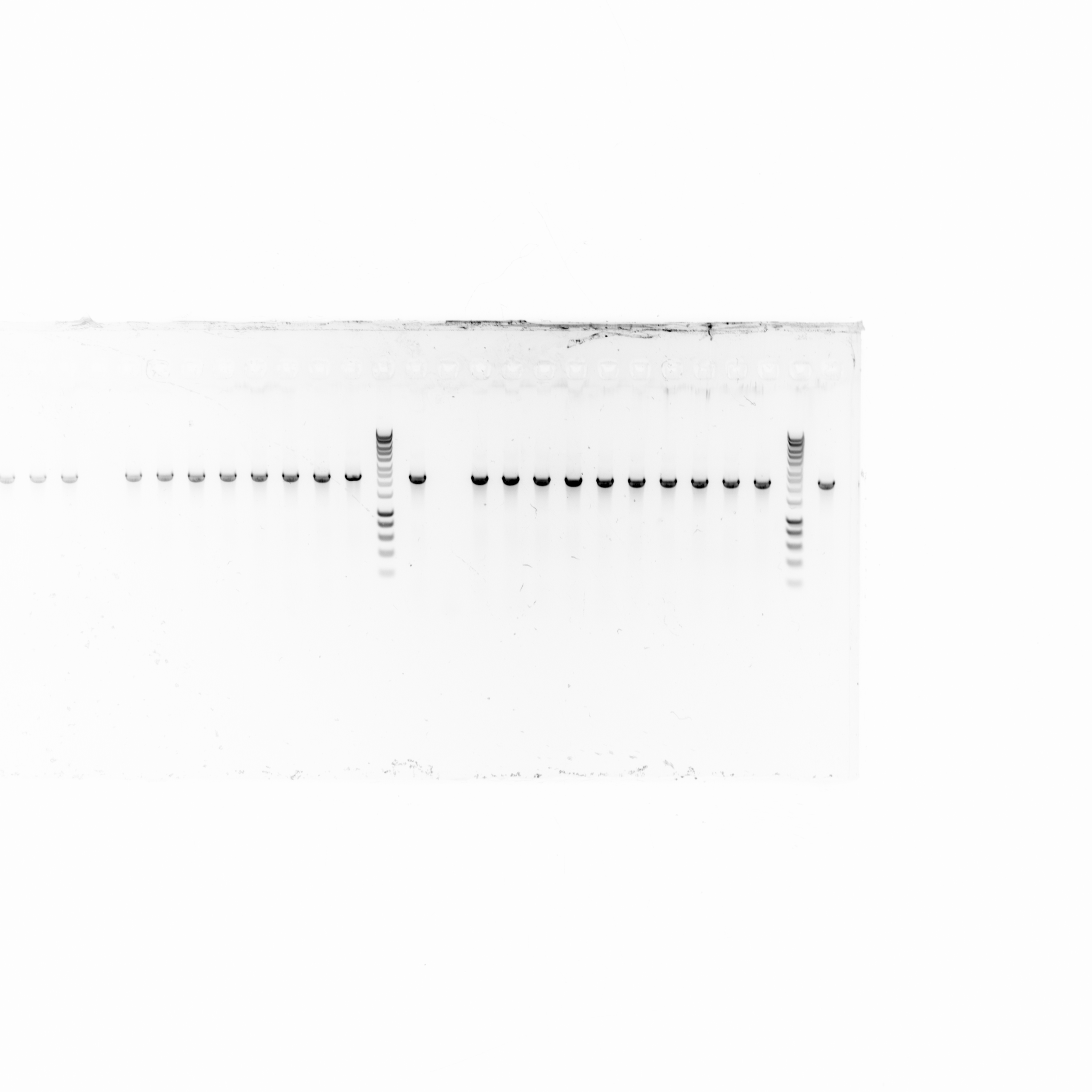

Supplement: Figure 4—source data 1. — The red box indicates the region of the gel used in the final figure. The lanes and identity of the band(s) are indicated. [file elife-84327-fig4-data1.zip › Figure 4 - Source data 1/Figure 4 - Source data 1 unedited.tif]

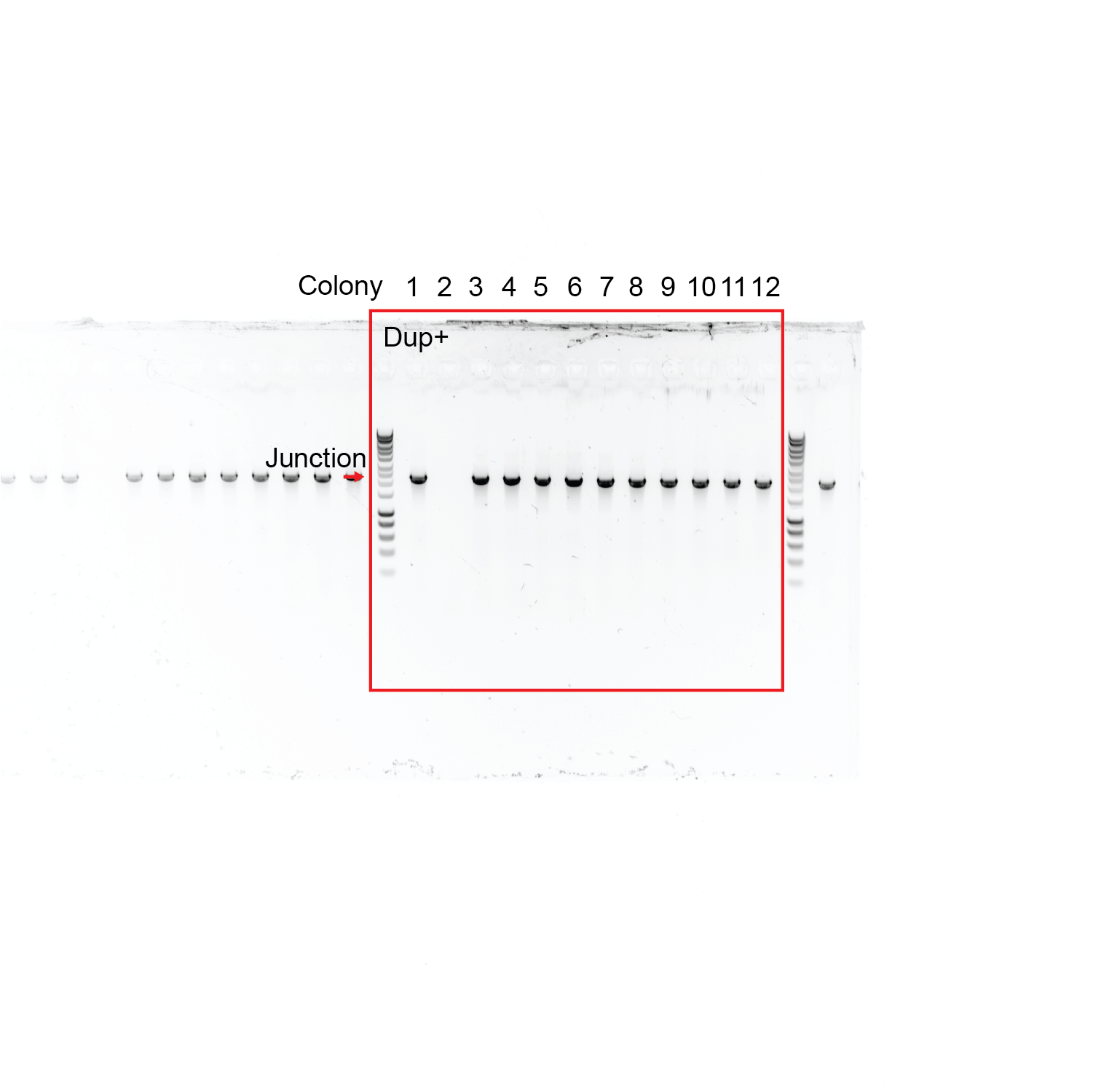

Supplement: Figure 4—source data 1. — The red box indicates the region of the gel used in the final figure. The lanes and identity of the band(s) are indicated. [file elife-84327-fig4-data1.zip › Figure 4 - Source data 1/Figure 4 - Source data 1.png]

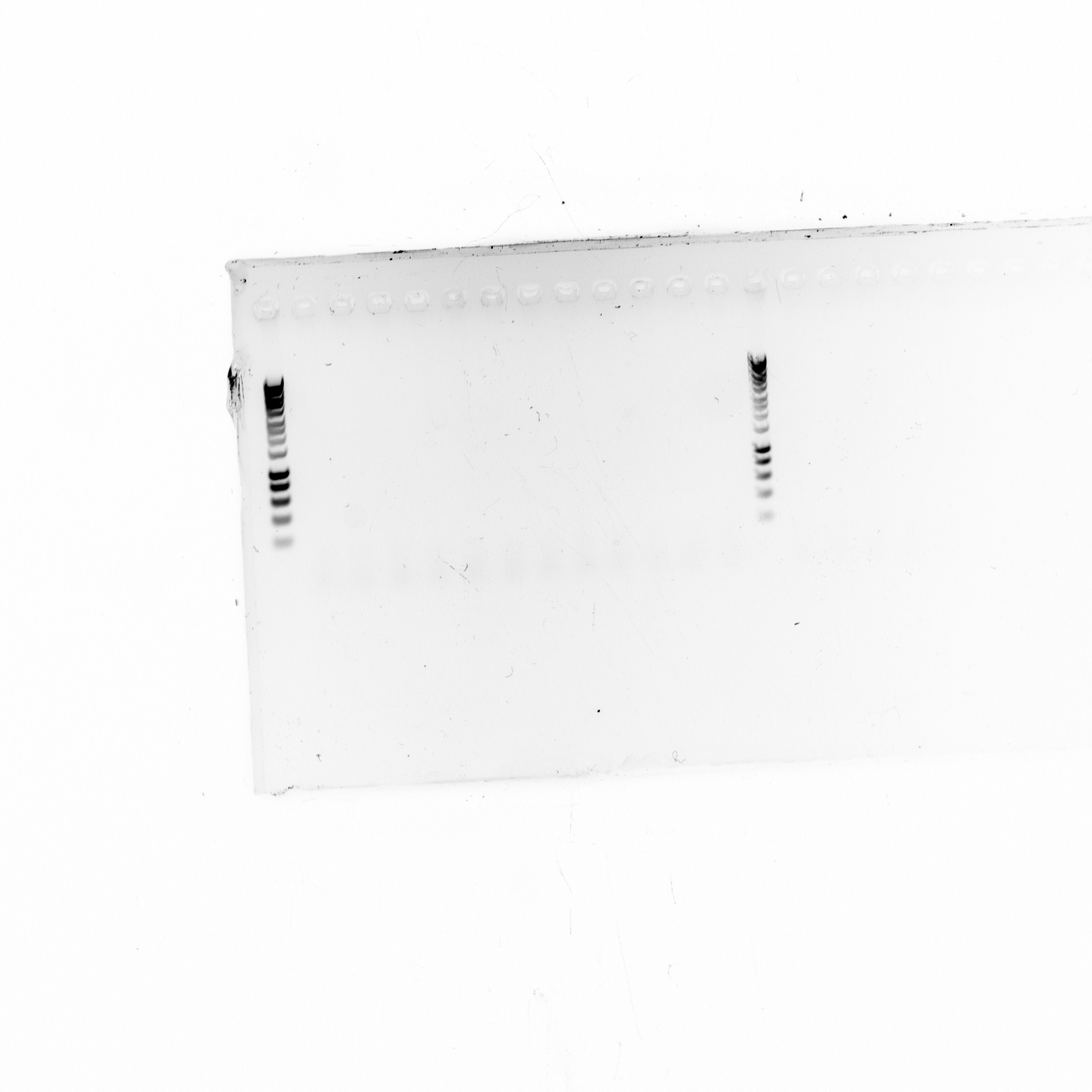

Supplement: Figure 4—source data 2. — The red box indicates the region of the gel used in the final figure. The lanes and identity of the band(s) are indicated. [file elife-84327-fig4-data2.zip › Figure 4 - Source data 2/Figure 4 - Source data 2 unedited.tif]

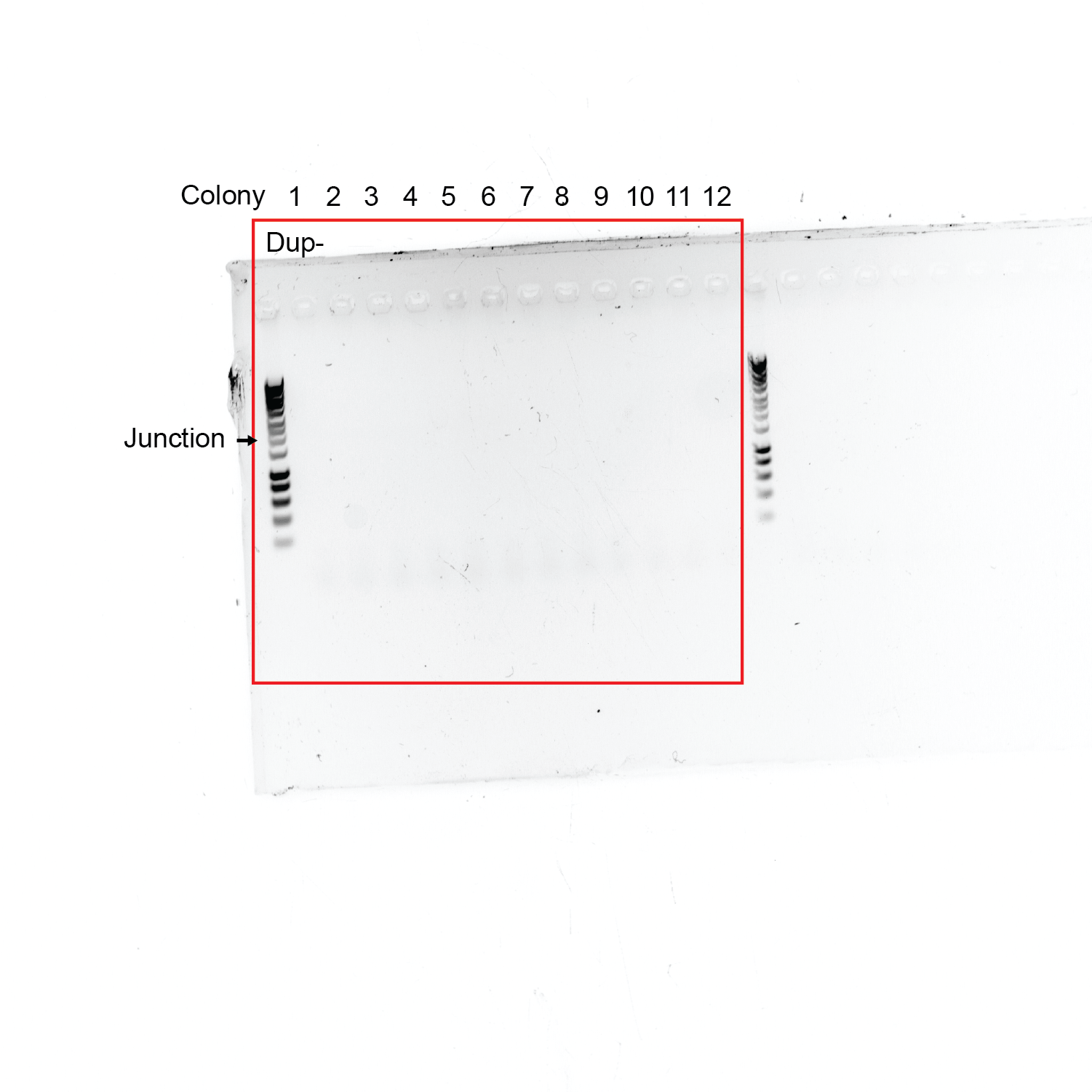

Supplement: Figure 4—source data 2. — The red box indicates the region of the gel used in the final figure. The lanes and identity of the band(s) are indicated. [file elife-84327-fig4-data2.zip › Figure 4 - Source data 2/Figure 4 - Source data 2.png]

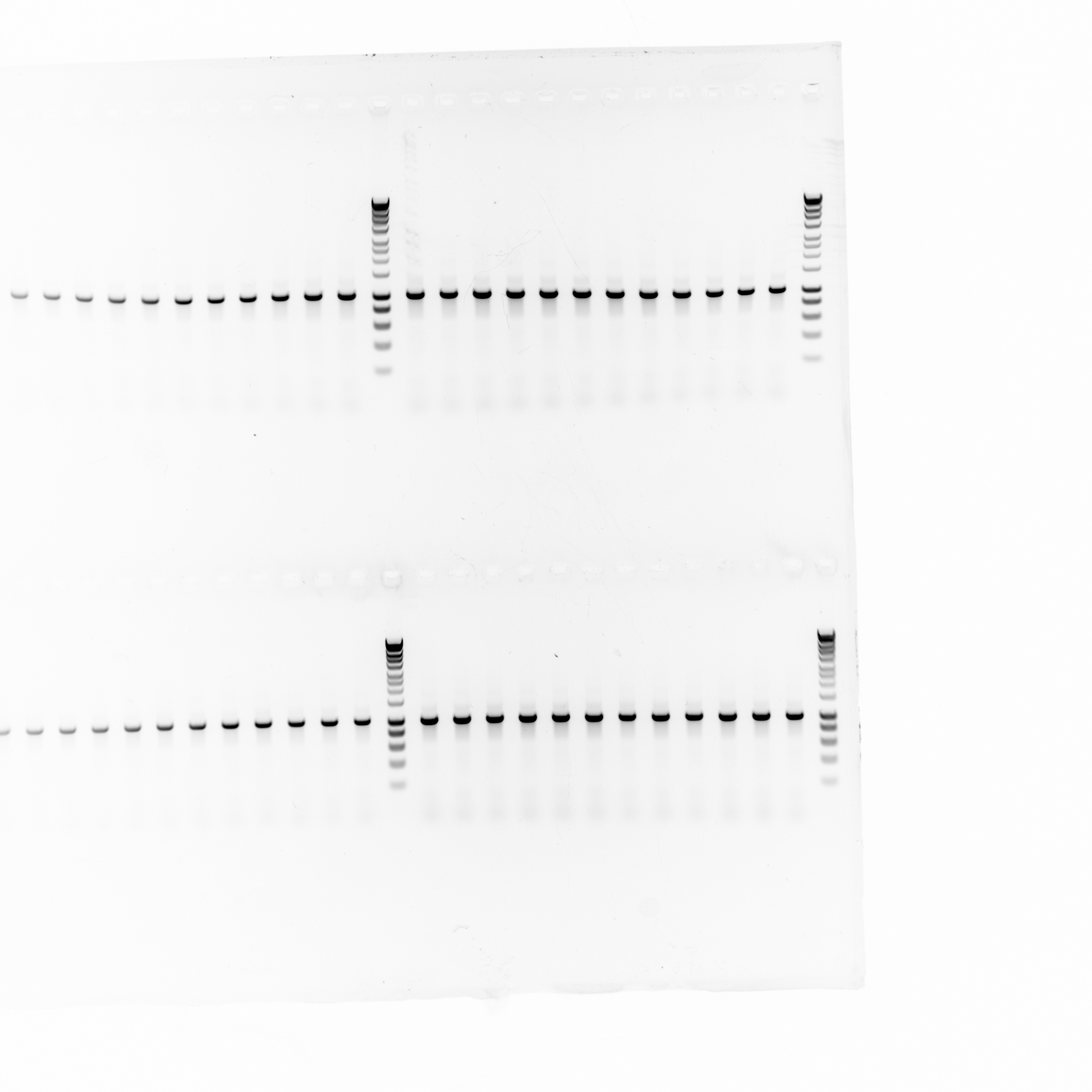

Supplement: Figure 4—source data 3. — The red box indicates the region of the gel used in the final figure. The lanes and identity of the band(s) are indicated. [file elife-84327-fig4-data3.zip › Figure 4 - Source data 3/Figure 4 - Source data 3 unedited.tif]

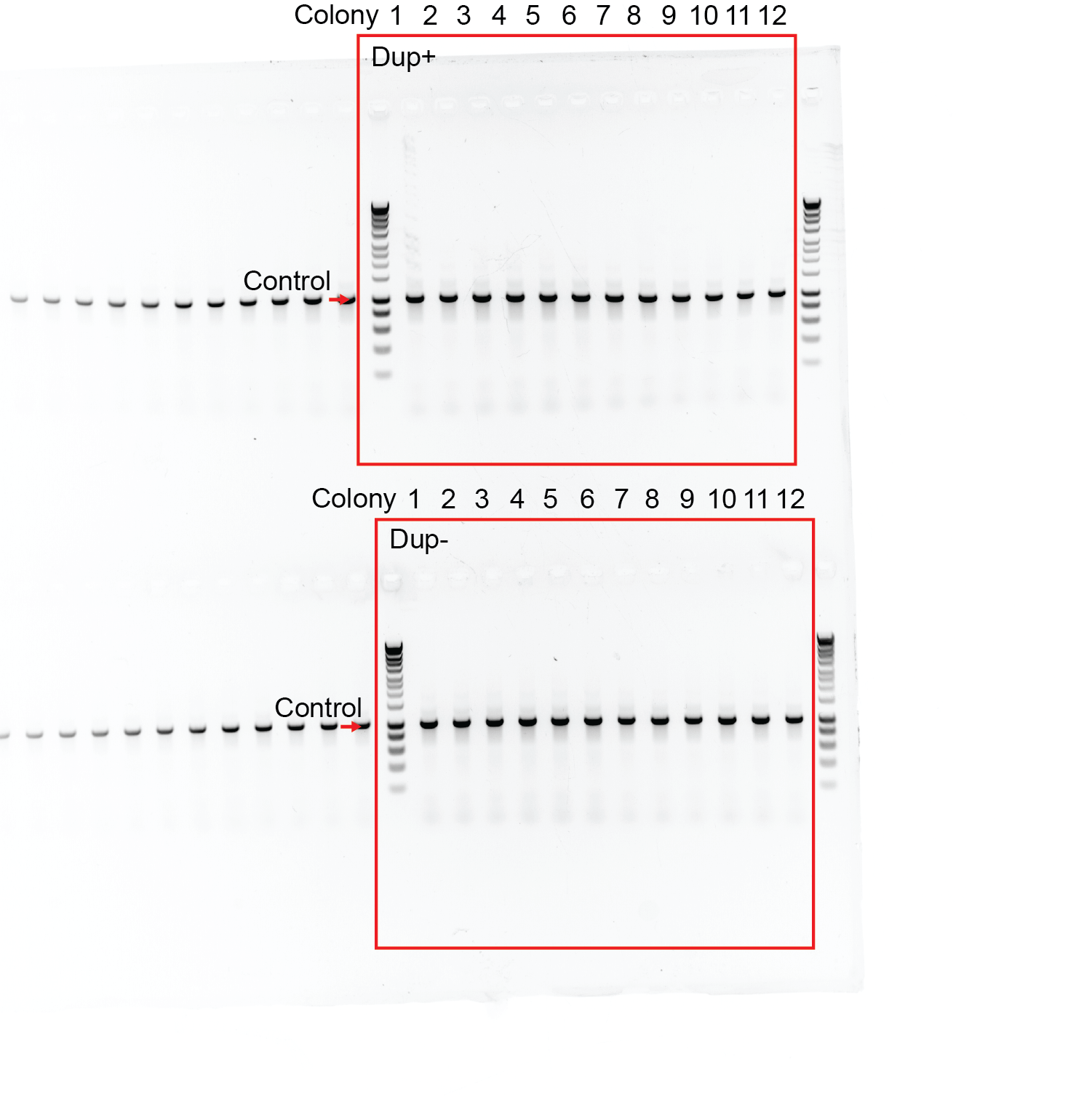

Supplement: Figure 4—source data 3. — The red box indicates the region of the gel used in the final figure. The lanes and identity of the band(s) are indicated. [file elife-84327-fig4-data3.zip › Figure 4 - Source data 3/Figure 4 - Source data 3.png]

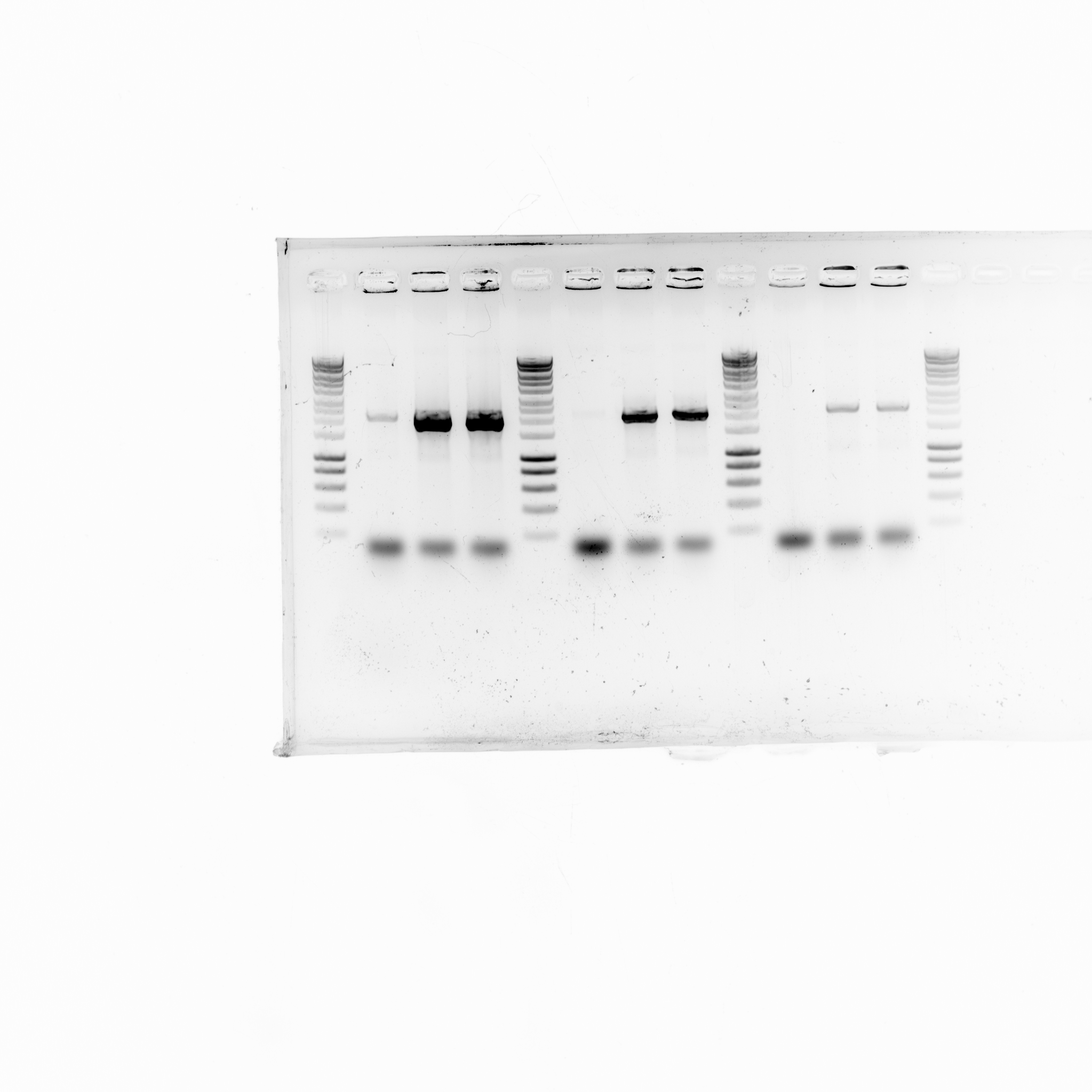

Supplement: Figure 5—source data 1. — The red box indicates the region of the gel used in the final figure. The lanes and identity of the band(s) are indicated. [file elife-84327-fig5-data1.zip › Figure 5 - Source data 1/Figure 5 - Source data 1 unedited.tif]

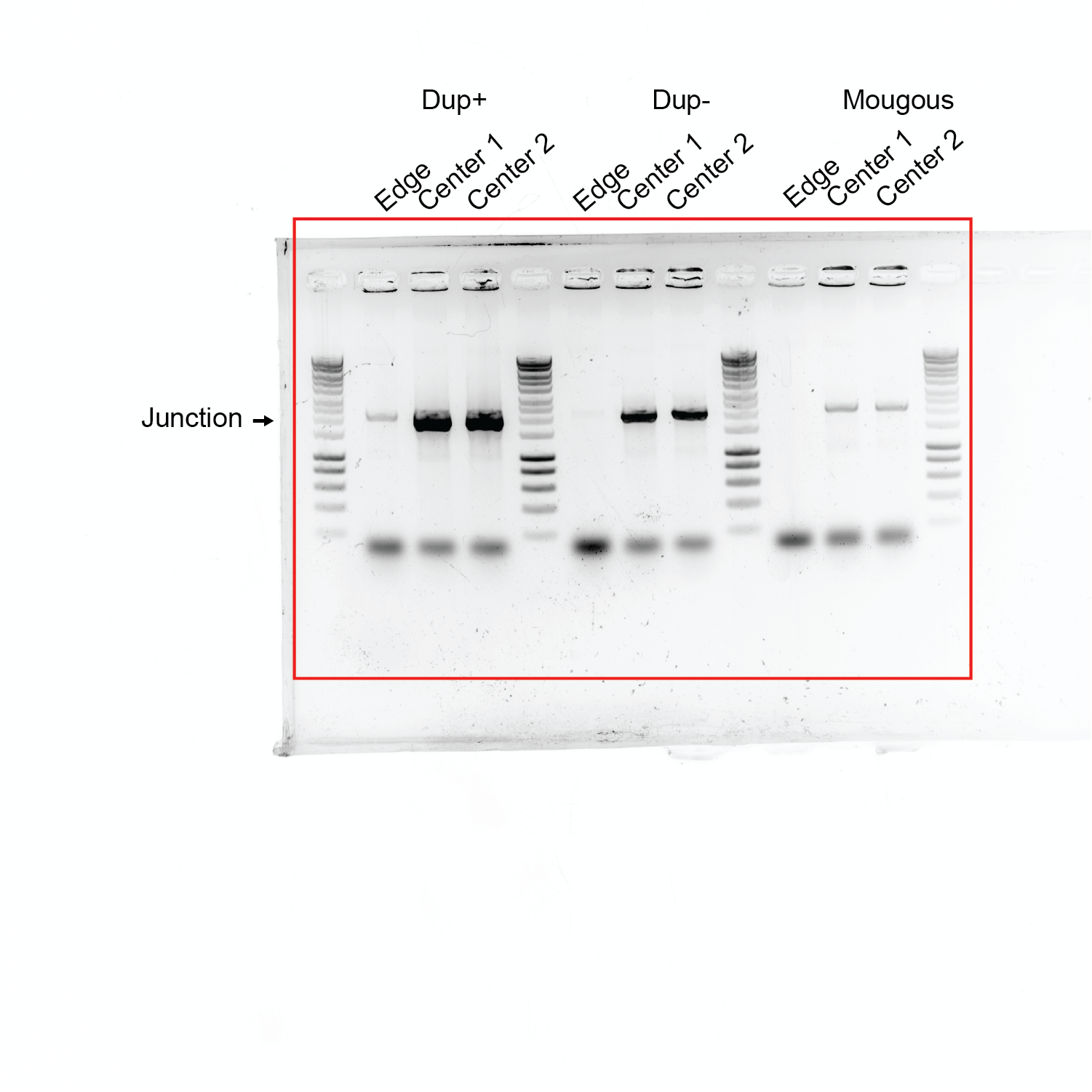

Supplement: Figure 5—source data 1. — The red box indicates the region of the gel used in the final figure. The lanes and identity of the band(s) are indicated. [file elife-84327-fig5-data1.zip › Figure 5 - Source data 1/Figure 5 - Source data 1.png]

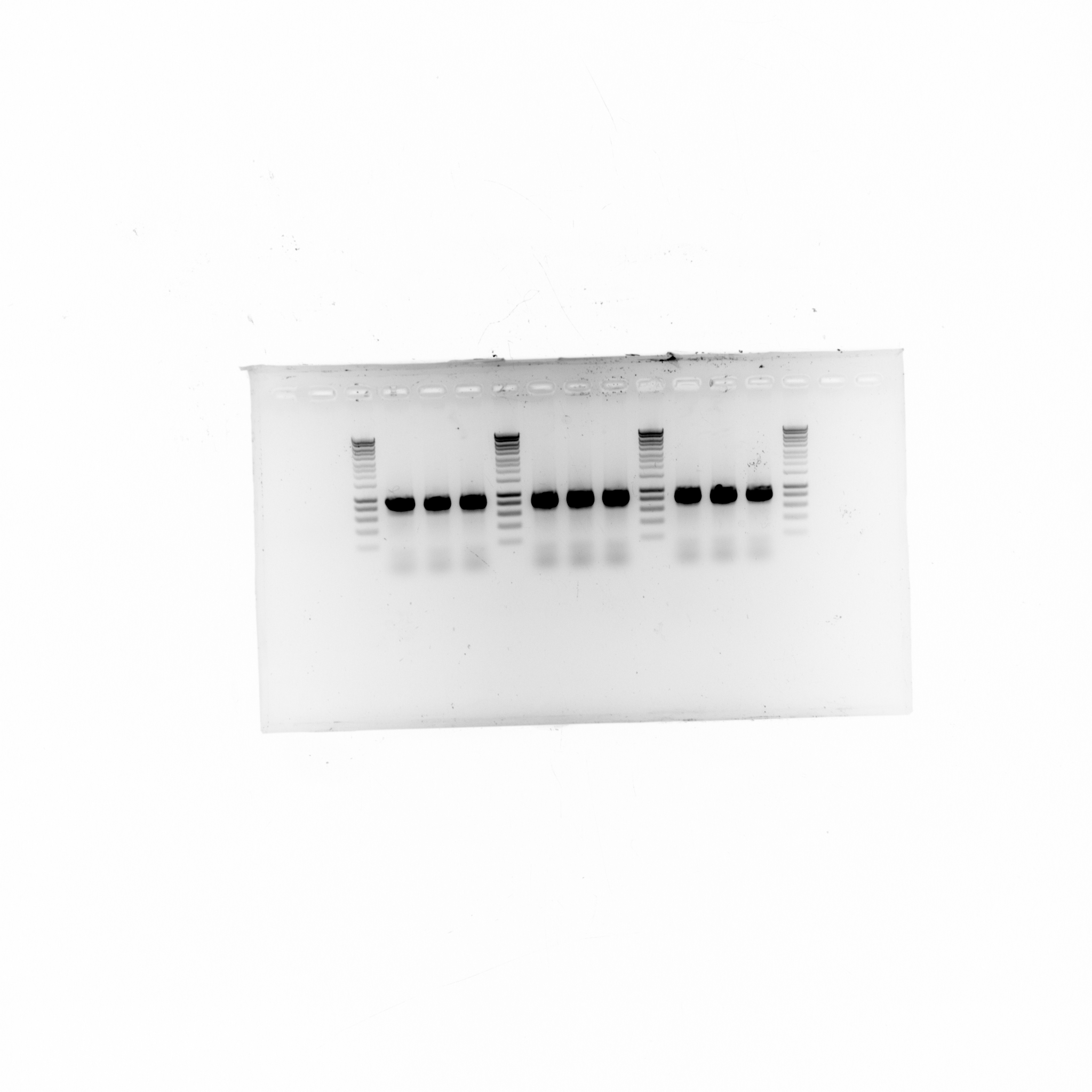

Supplement: Figure 5—source data 2. — The red box indicates the region of the gel used in the final figure. The lanes and identity of the band(s) are indicated. [file elife-84327-fig5-data2.zip › Figure 5 - Source data 2/Figure 5 - Source data 2 unedited.png]

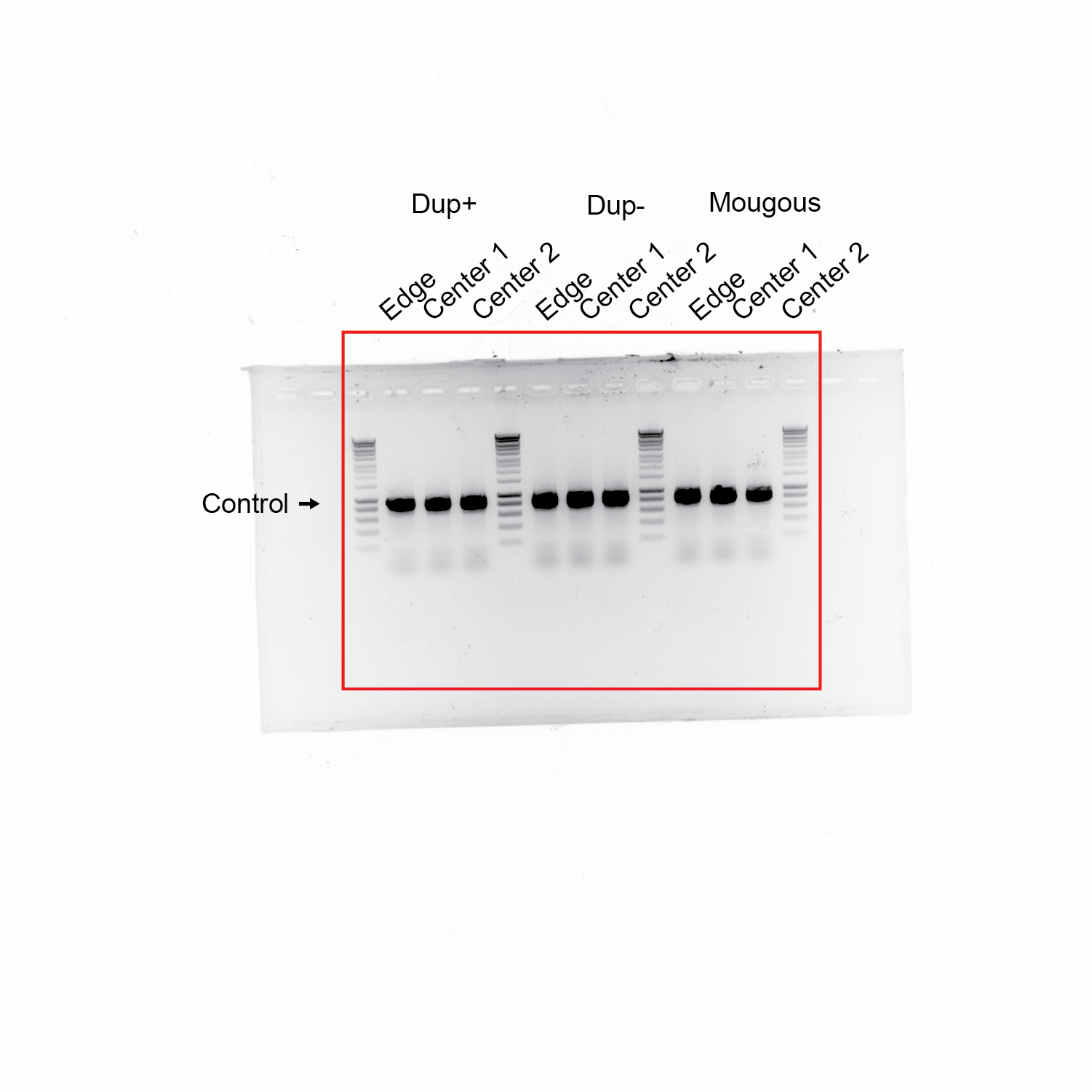

Supplement: Figure 5—source data 2. — The red box indicates the region of the gel used in the final figure. The lanes and identity of the band(s) are indicated. [file elife-84327-fig5-data2.zip › Figure 5 - Source data 2/Figure 5 - Source data 2.png]

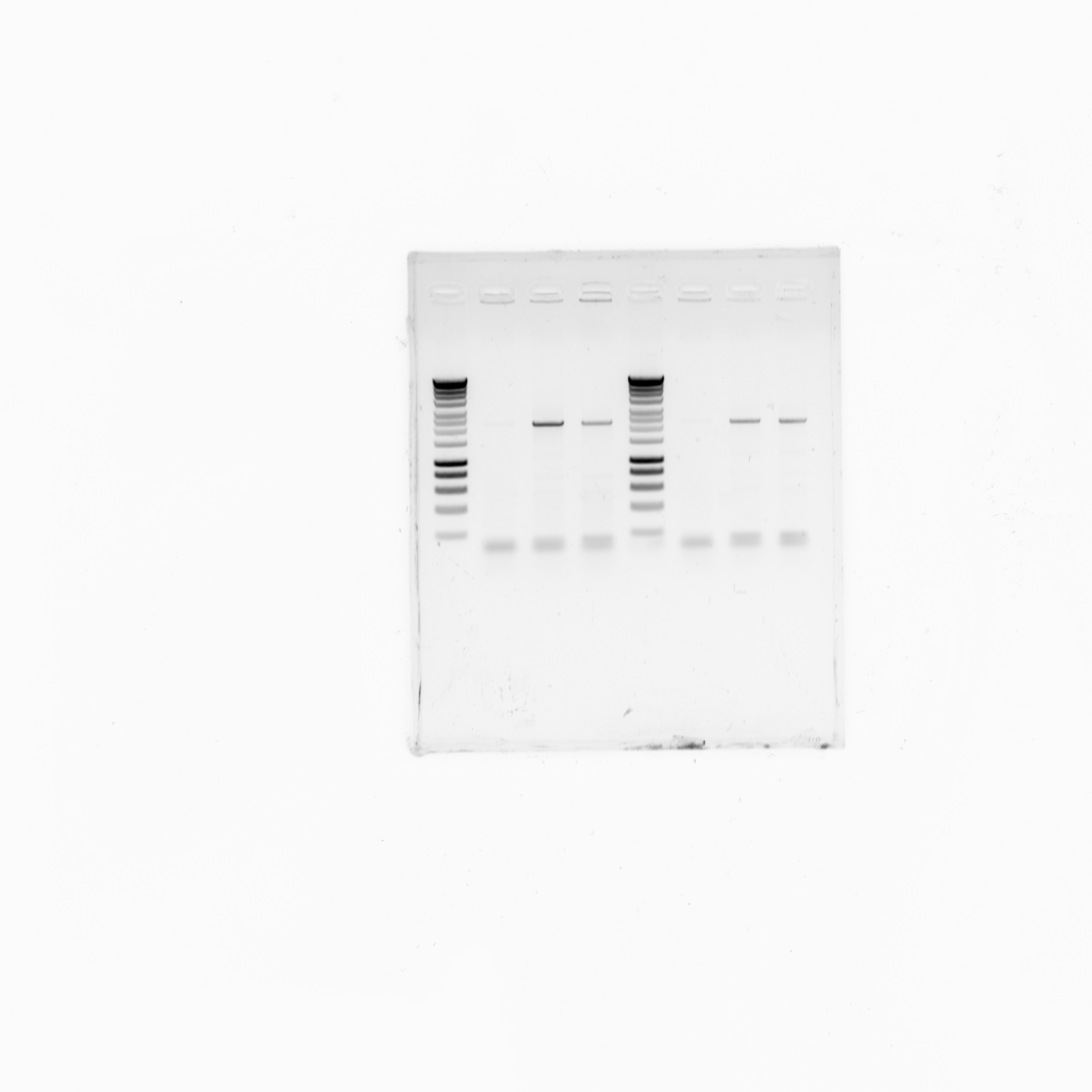

Supplement: Figure 5—source data 3. — The red box indicates the region of the gel used in the final figure. The lanes and identity of the band(s) are indicated. [file elife-84327-fig5-data3.zip › Figure 5 - Source data 3/Fig 5 - Source data 3 unedited.tif]

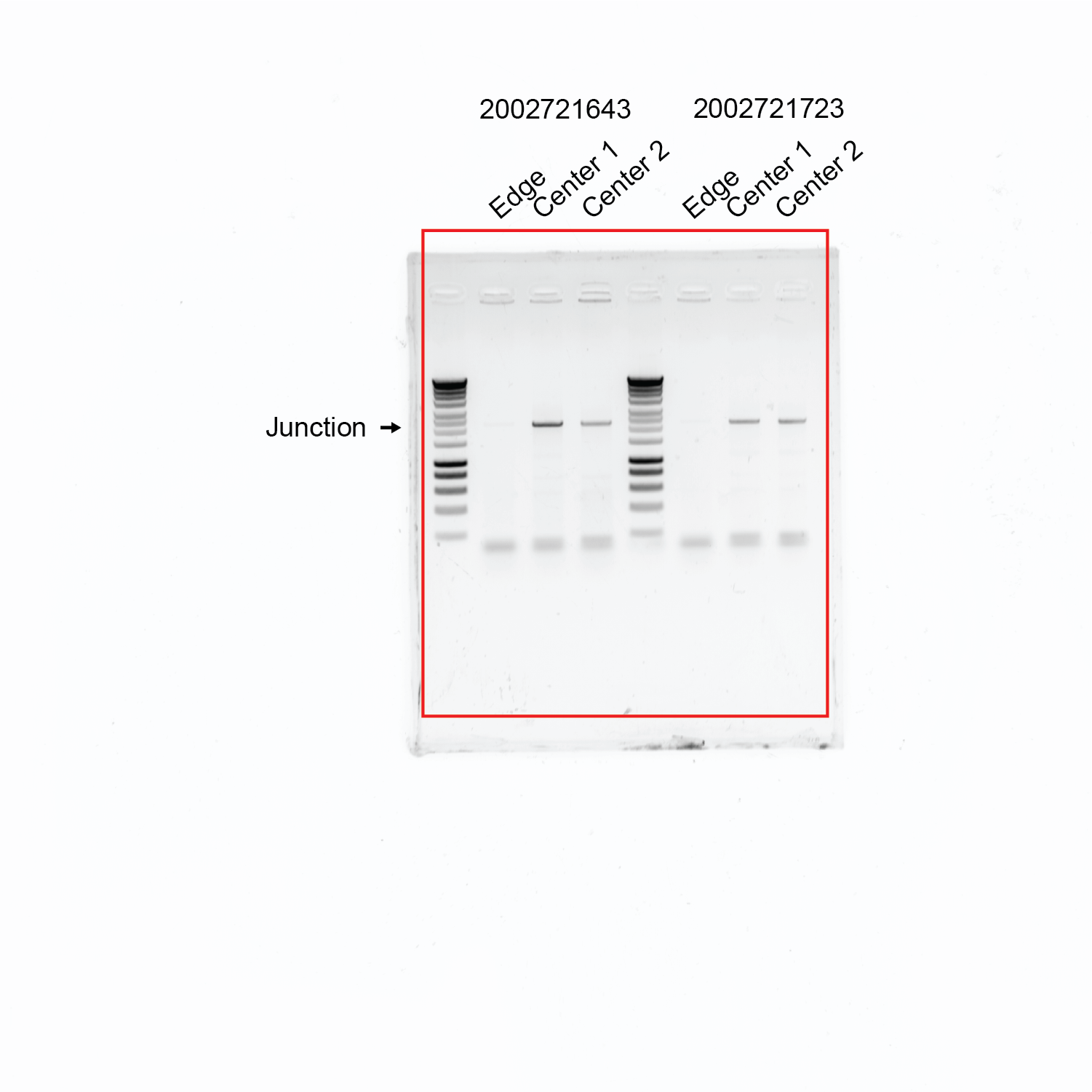

Supplement: Figure 5—source data 3. — The red box indicates the region of the gel used in the final figure. The lanes and identity of the band(s) are indicated. [file elife-84327-fig5-data3.zip › Figure 5 - Source data 3/Figure 5 - Source data 3.png]

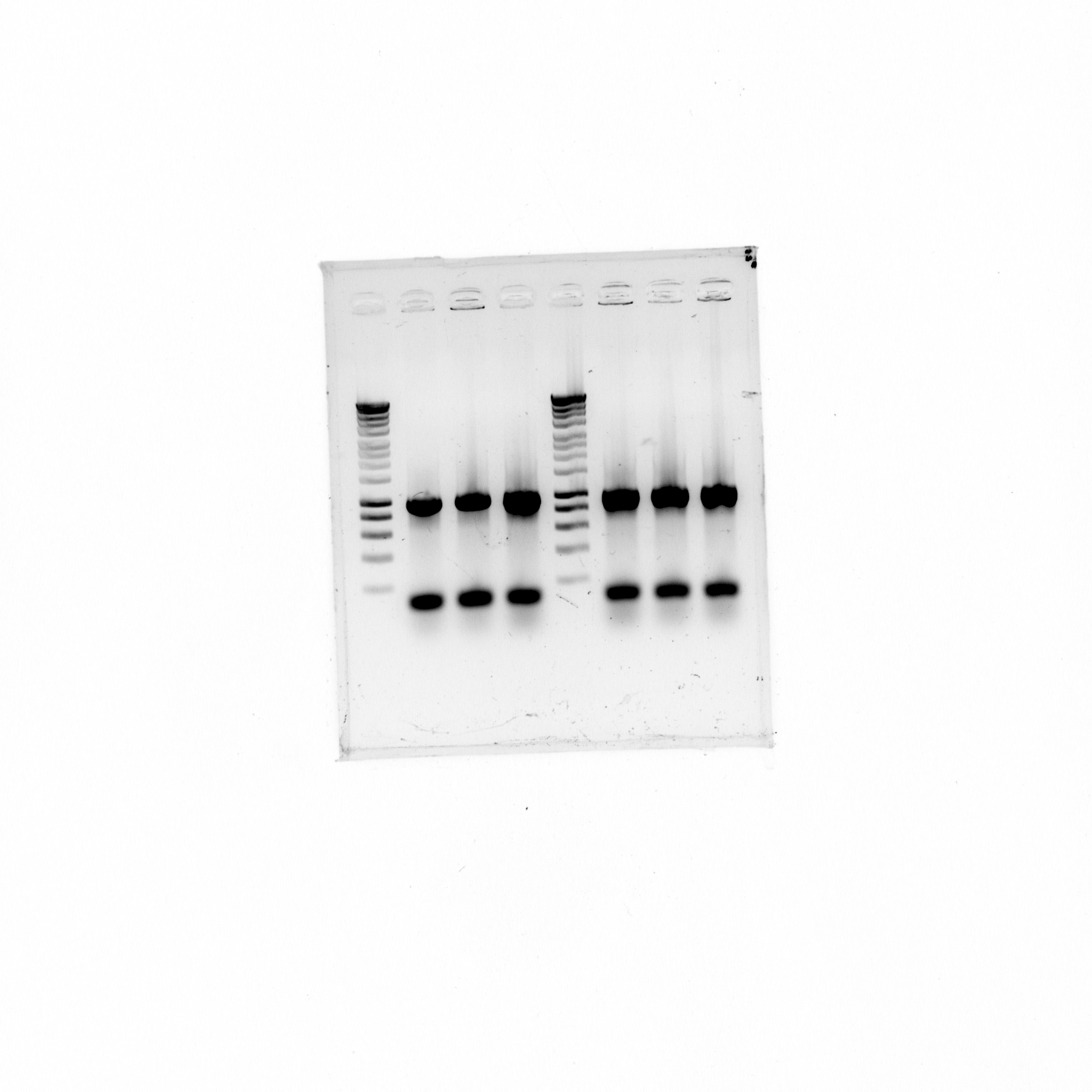

Supplement: Figure 5—source data 4. — The red box indicates the region of the gel used in the final figure. The lanes and identity of the band(s) are indicated. [file elife-84327-fig5-data4.zip › Figure 5 - Source data 4/Fig 5 - Source data 4 unedited.tif]

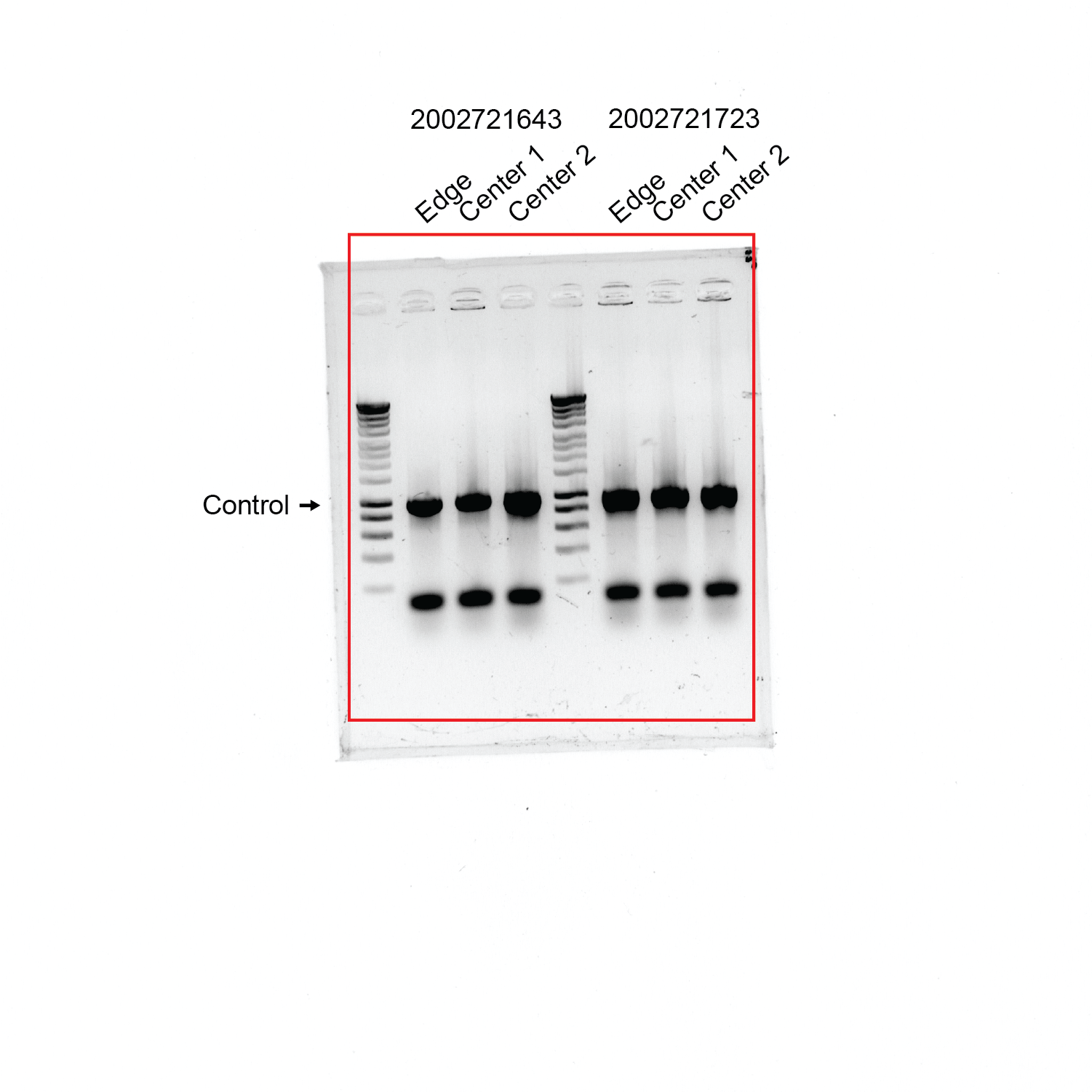

Supplement: Figure 5—source data 4. — The red box indicates the region of the gel used in the final figure. The lanes and identity of the band(s) are indicated. [file elife-84327-fig5-data4.zip › Figure 5 - Source data 4/Figure 5 - Source data 4.png]

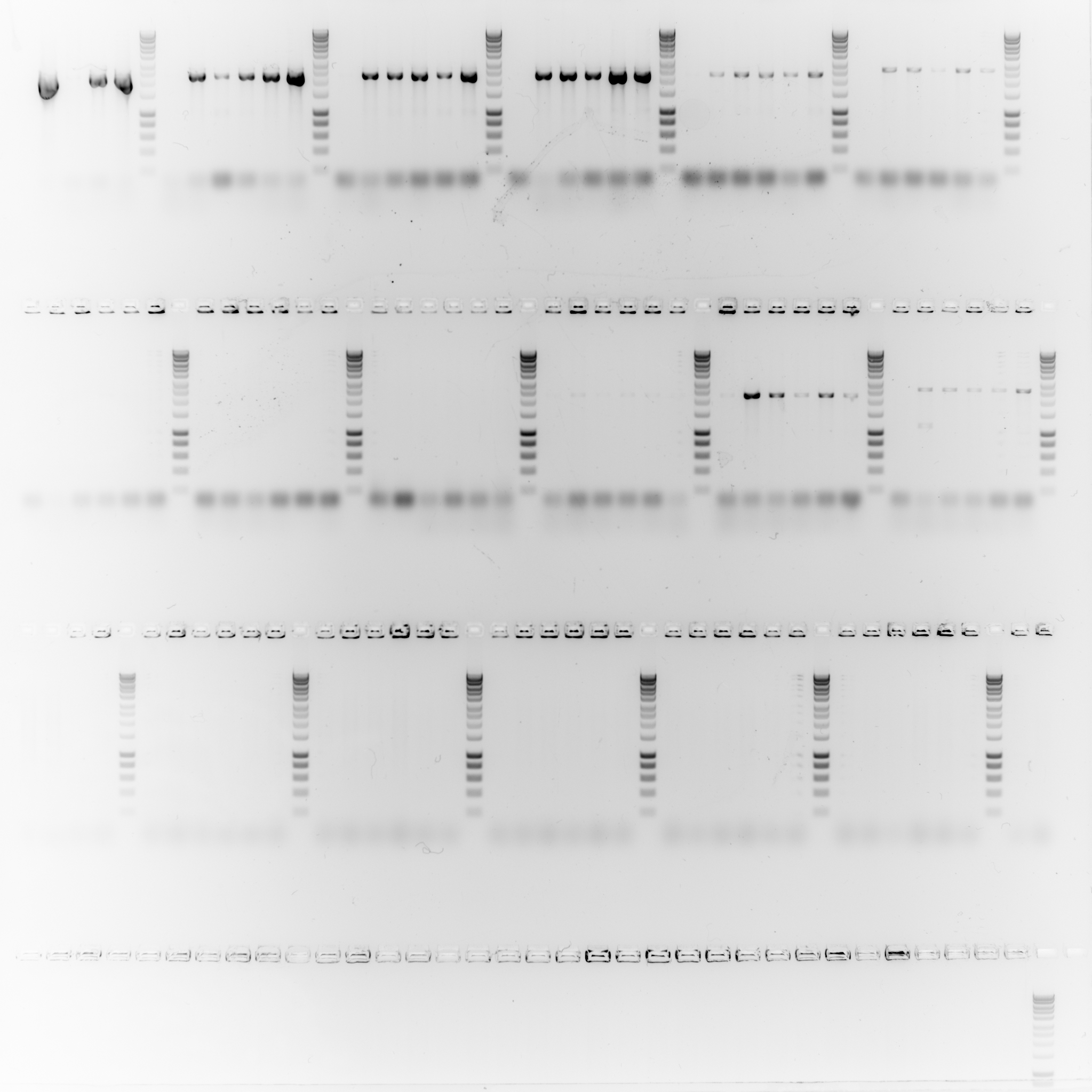

Supplement: Figure 6—source data 1. — The red box indicates the region of the gel used in the final figure. The lanes and identity of the band(s) are indicated. [file elife-84327-fig6-data1.zip › Figure 6 - Source data 1/Figure 6 - Source data 1 unedited.tif]

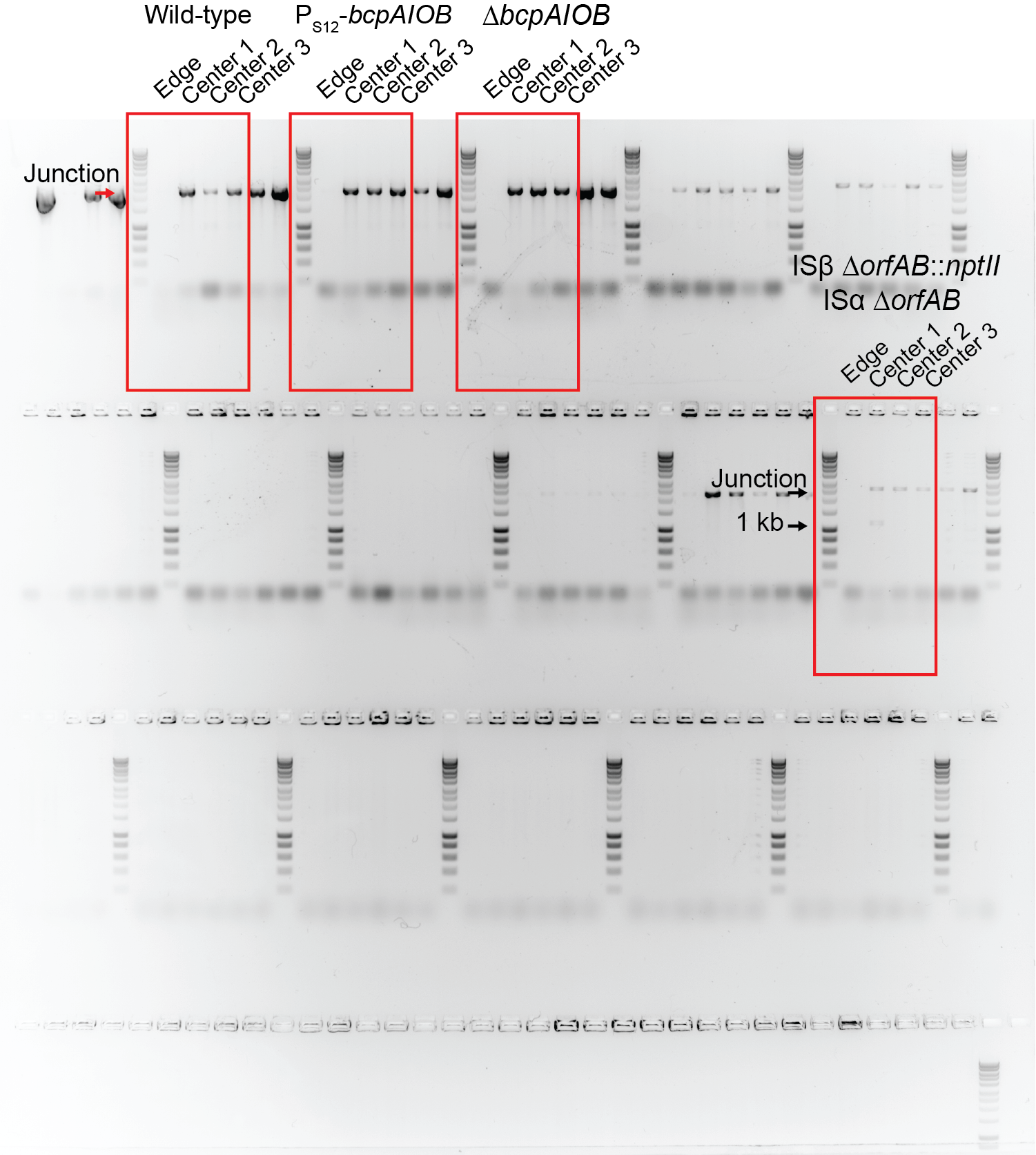

Supplement: Figure 6—source data 1. — The red box indicates the region of the gel used in the final figure. The lanes and identity of the band(s) are indicated. [file elife-84327-fig6-data1.zip › Figure 6 - Source data 1/Figure 6 - Source data 1.png]

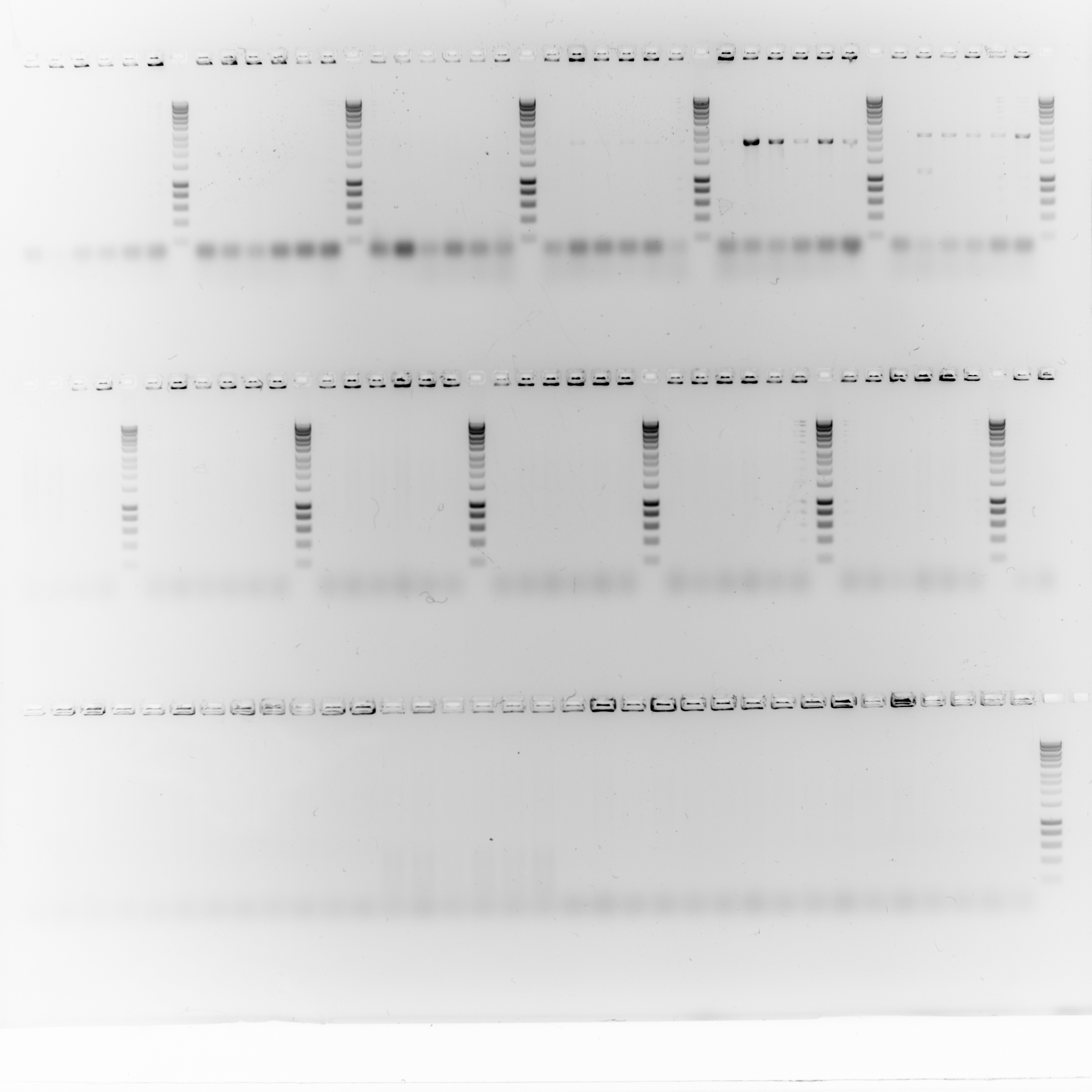

Supplement: Figure 6—source data 2. — The red box indicates the region of the gel used in the final figure. The lanes and identity of the band(s) are indicated. [file elife-84327-fig6-data2.zip › Figure 6 - Source data 2/Figure 6 - Source data 2 unedited.tif]

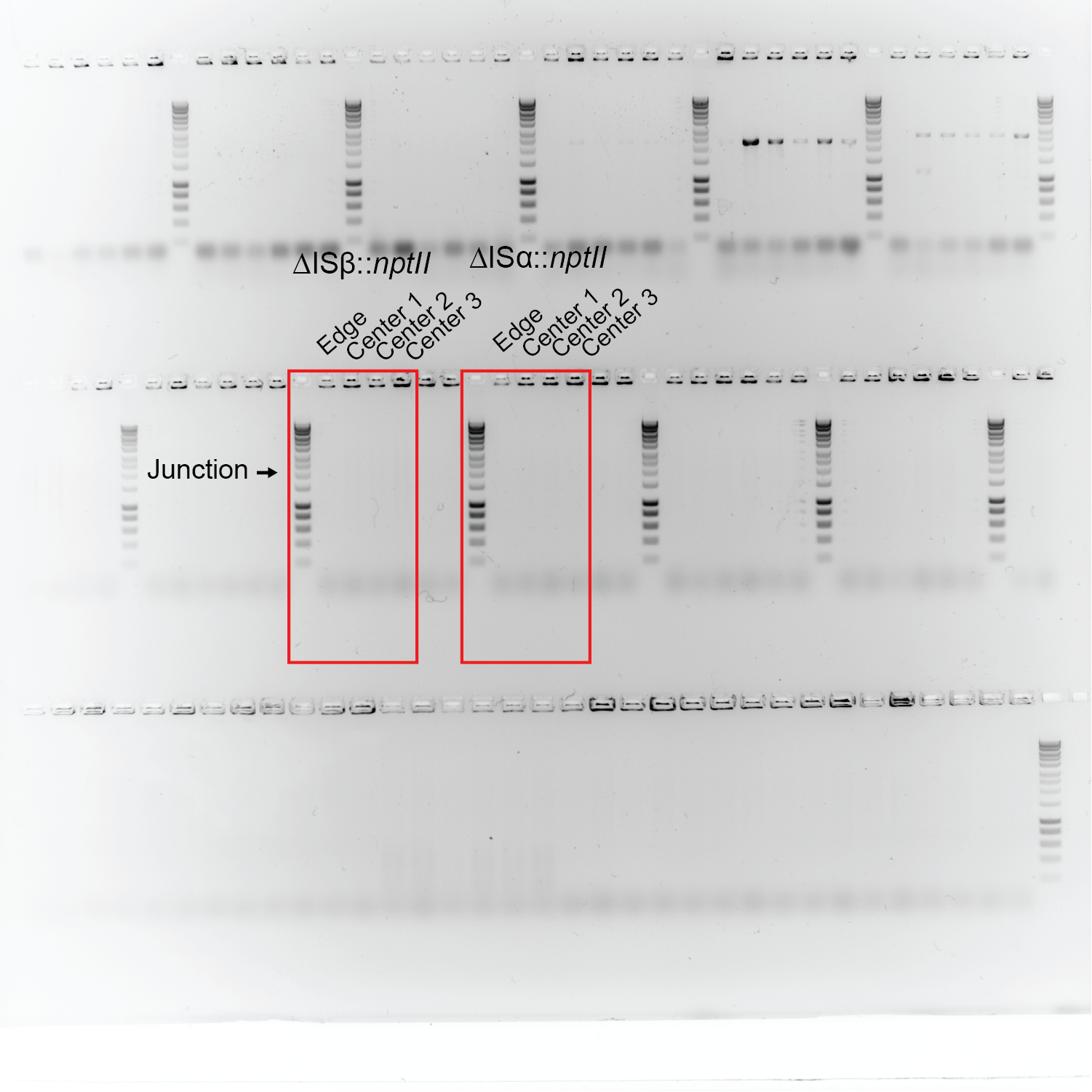

Supplement: Figure 6—source data 2. — The red box indicates the region of the gel used in the final figure. The lanes and identity of the band(s) are indicated. [file elife-84327-fig6-data2.zip › Figure 6 - Source data 2/Figure 6 - Source data 2.png]

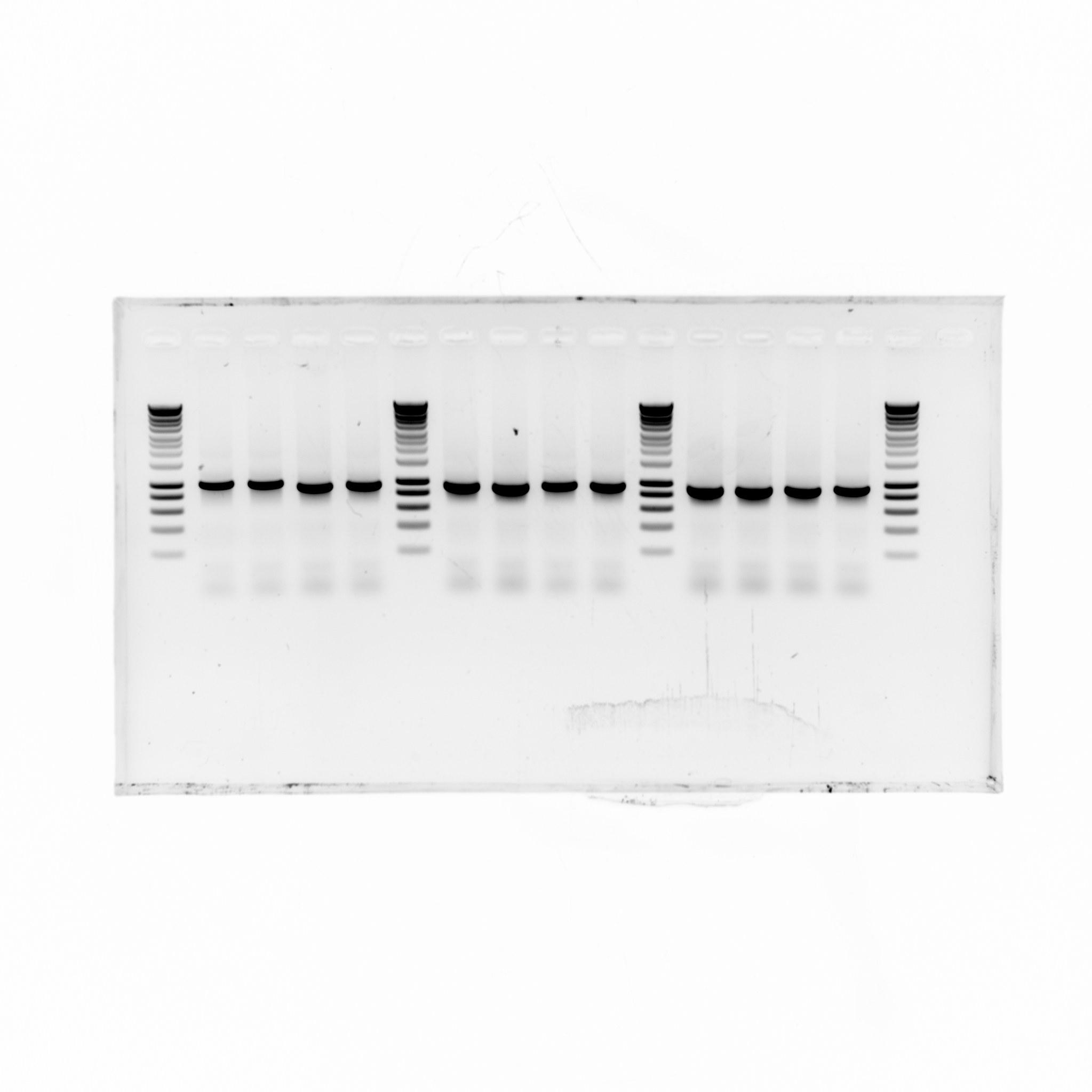

Supplement: Figure 6—source data 3. — The red box indicates the region of the gel used in the final figure. The lanes and identity of the band(s) are indicated. [file elife-84327-fig6-data3.zip › Figure 6 - Source data 3/Figure 6 - Source data 3 unedited.tif]

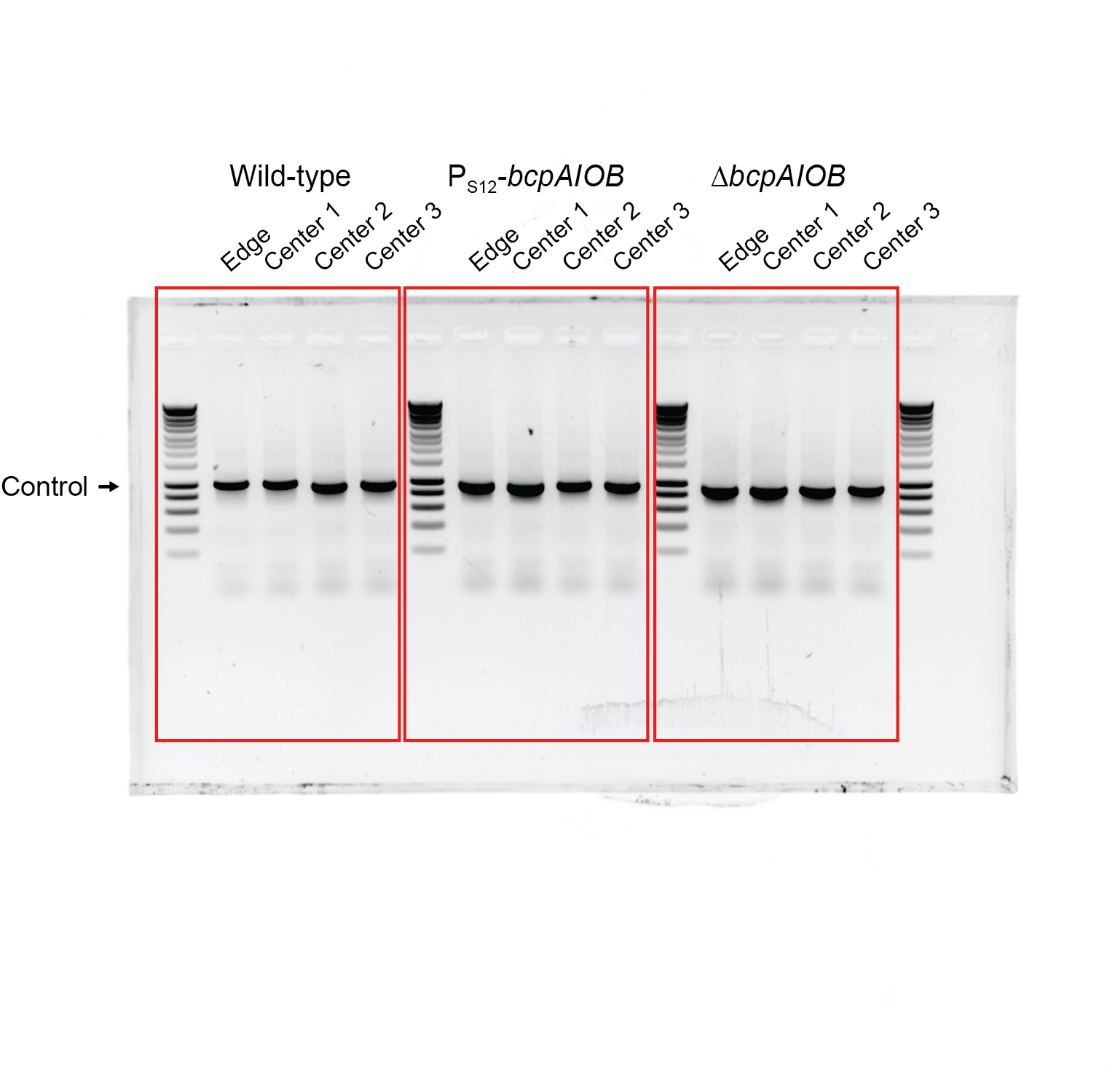

Supplement: Figure 6—source data 3. — The red box indicates the region of the gel used in the final figure. The lanes and identity of the band(s) are indicated. [file elife-84327-fig6-data3.zip › Figure 6 - Source data 3/Figure 6 - Source data 3.png]

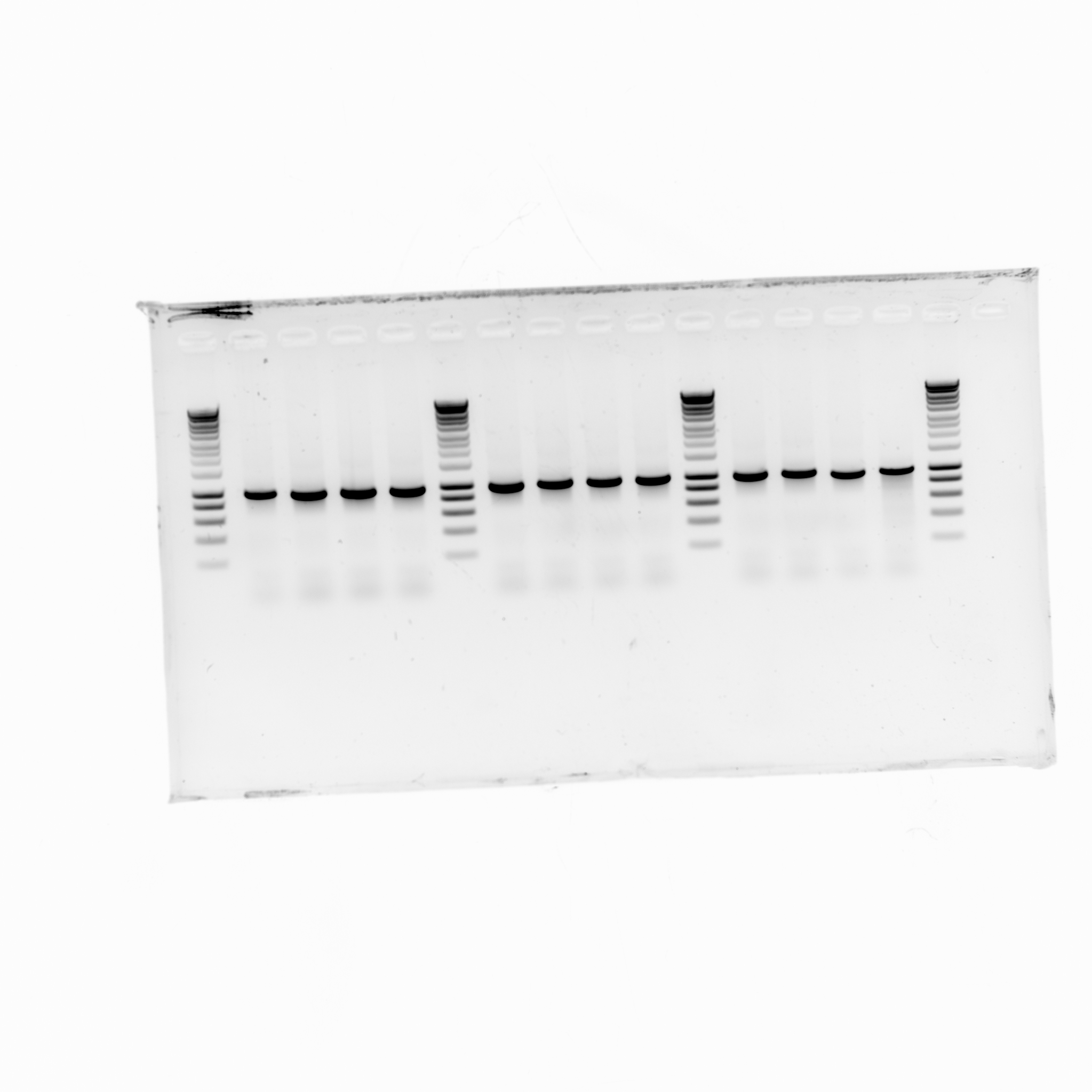

Supplement: Figure 6—source data 4. — The red box indicates the region of the gel used in the final figure. The lanes and identity of the band(s) are indicated. [file elife-84327-fig6-data4.zip › Figure 6 - Source data 4/Figure 6 - Source data 4 unedited.tif]

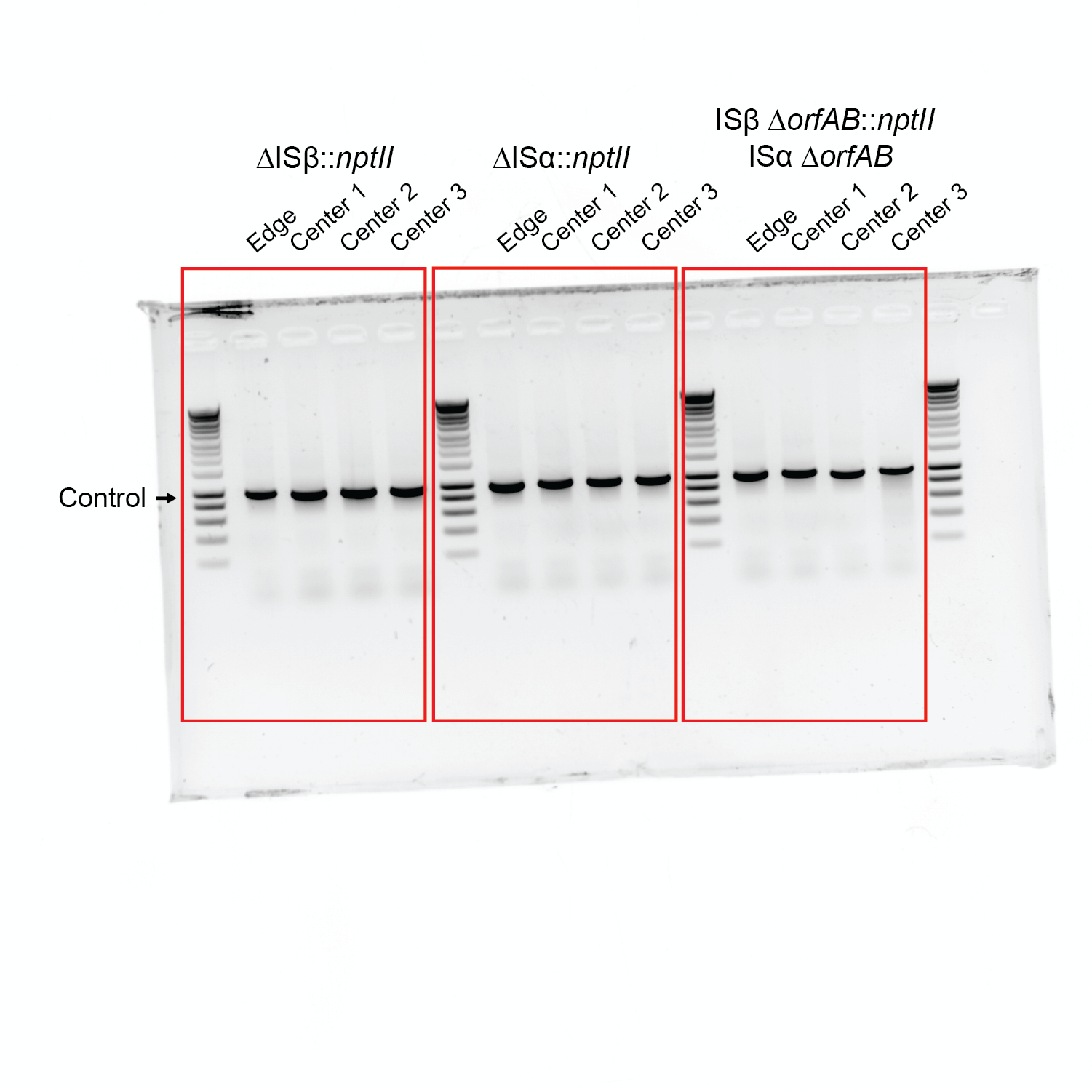

Supplement: Figure 6—source data 4. — The red box indicates the region of the gel used in the final figure. The lanes and identity of the band(s) are indicated. [file elife-84327-fig6-data4.zip › Figure 6 - Source data 4/Figure 6 - Source data 4.png]

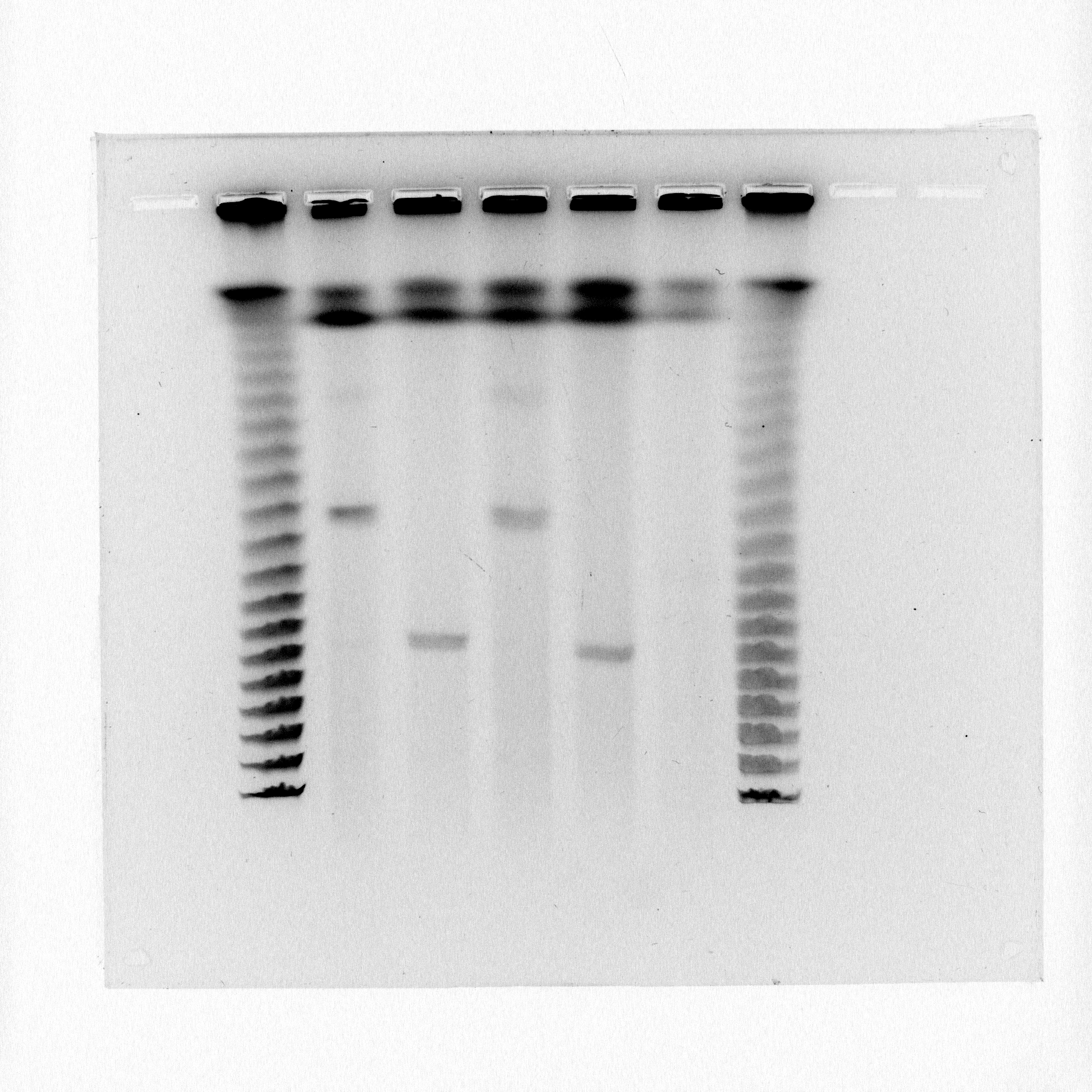

Supplement: Figure 6—figure supplement 1—source data 1. — The red box indicates the region of the gel used in the final figure. The lanes and identity of the band(s) are indicated. [file elife-84327-fig6-figsupp1-data1.zip › Figure 6 - Source data 5/Figure 6 - Source data 5 unedited.tif]

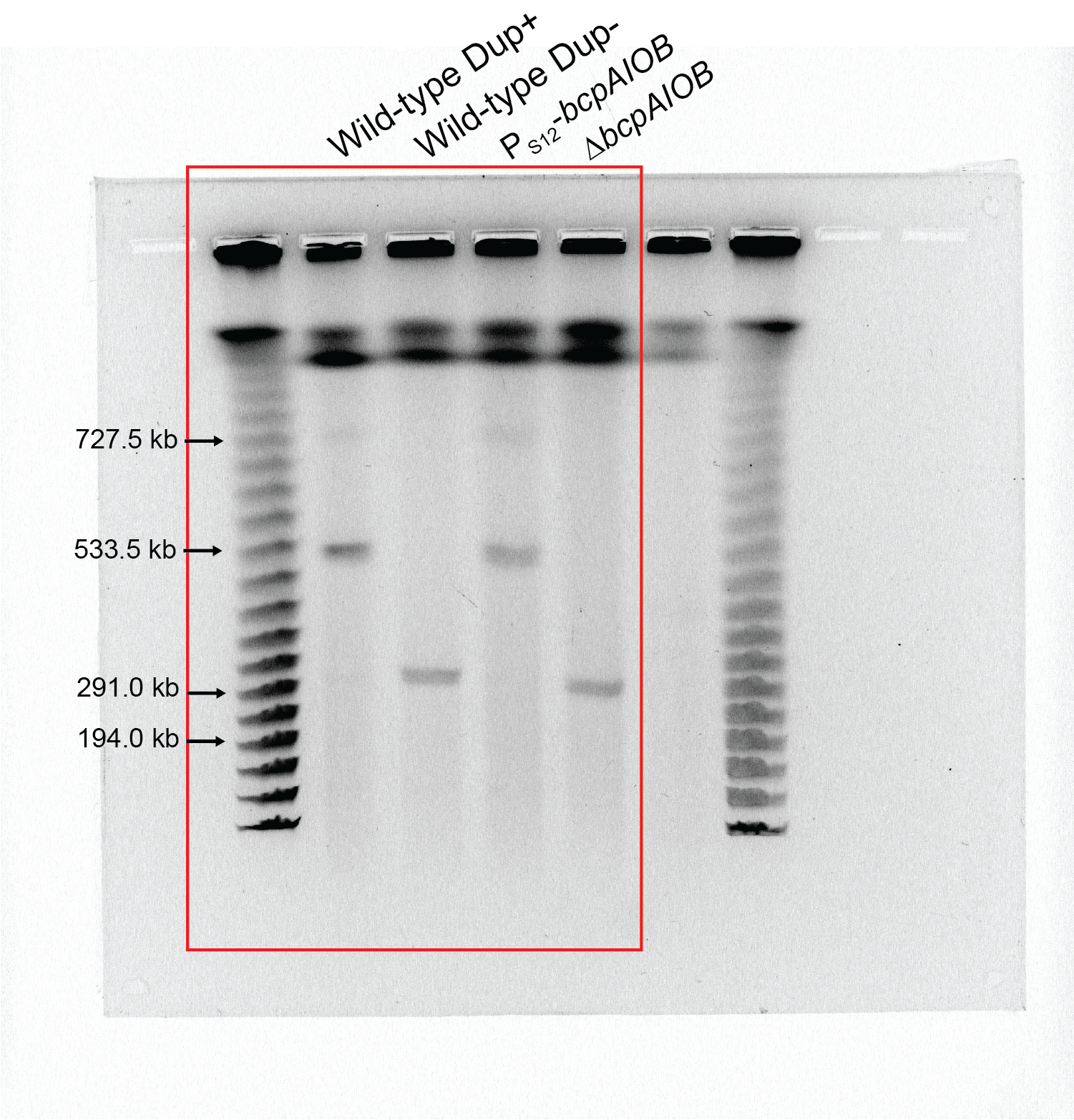

Supplement: Figure 6—figure supplement 1—source data 1. — The red box indicates the region of the gel used in the final figure. The lanes and identity of the band(s) are indicated. [file elife-84327-fig6-figsupp1-data1.zip › Figure 6 - Source data 5/Figure 6 - Source data 5.png]

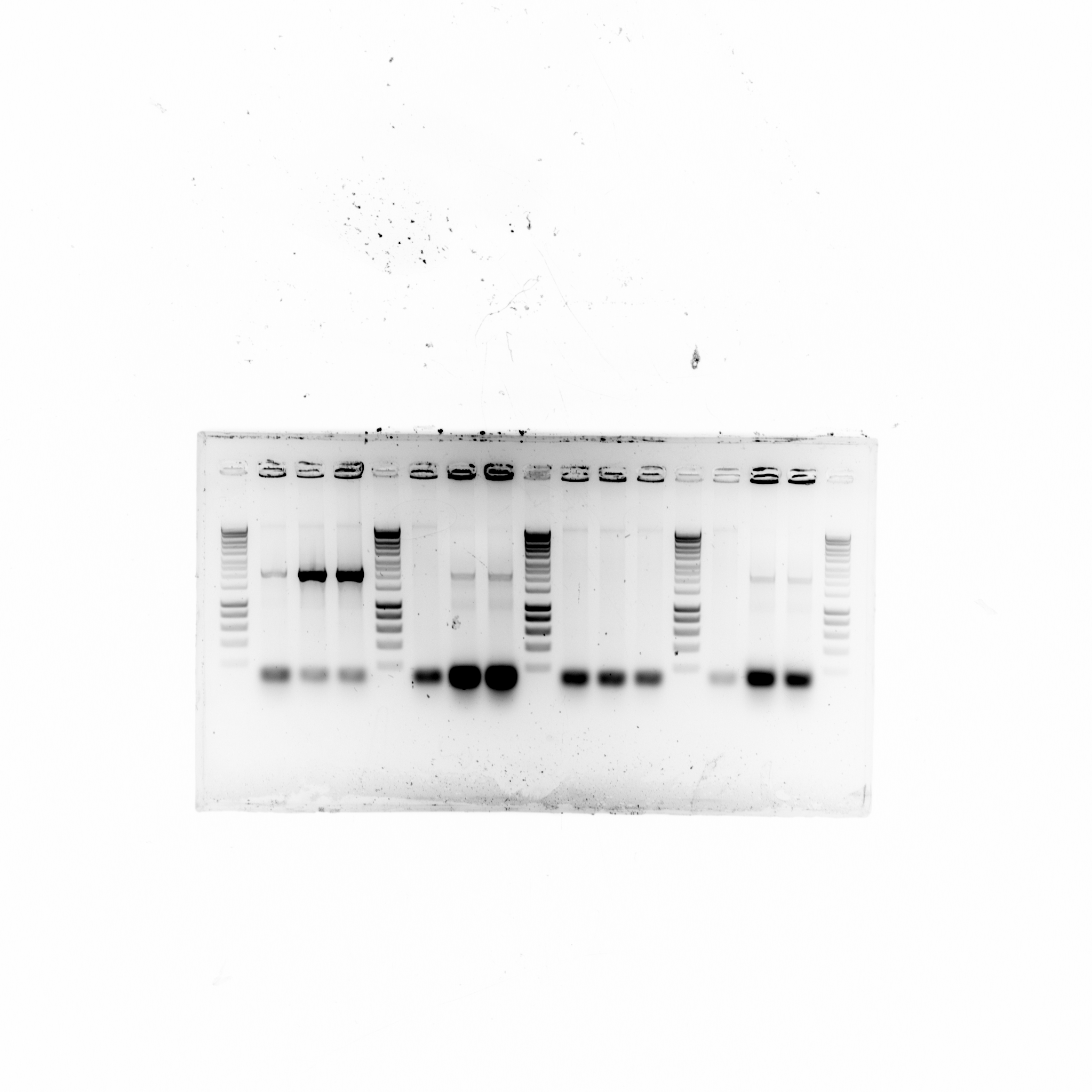

Supplement: Figure 7—source data 1. — The red box indicates the region of the gel used in the final figure. The lanes and identity of the band(s) are indicated. [file elife-84327-fig7-data1.zip › Figure 7 - Source data 1/Figure 7 - Source data 1 unedited.tif]

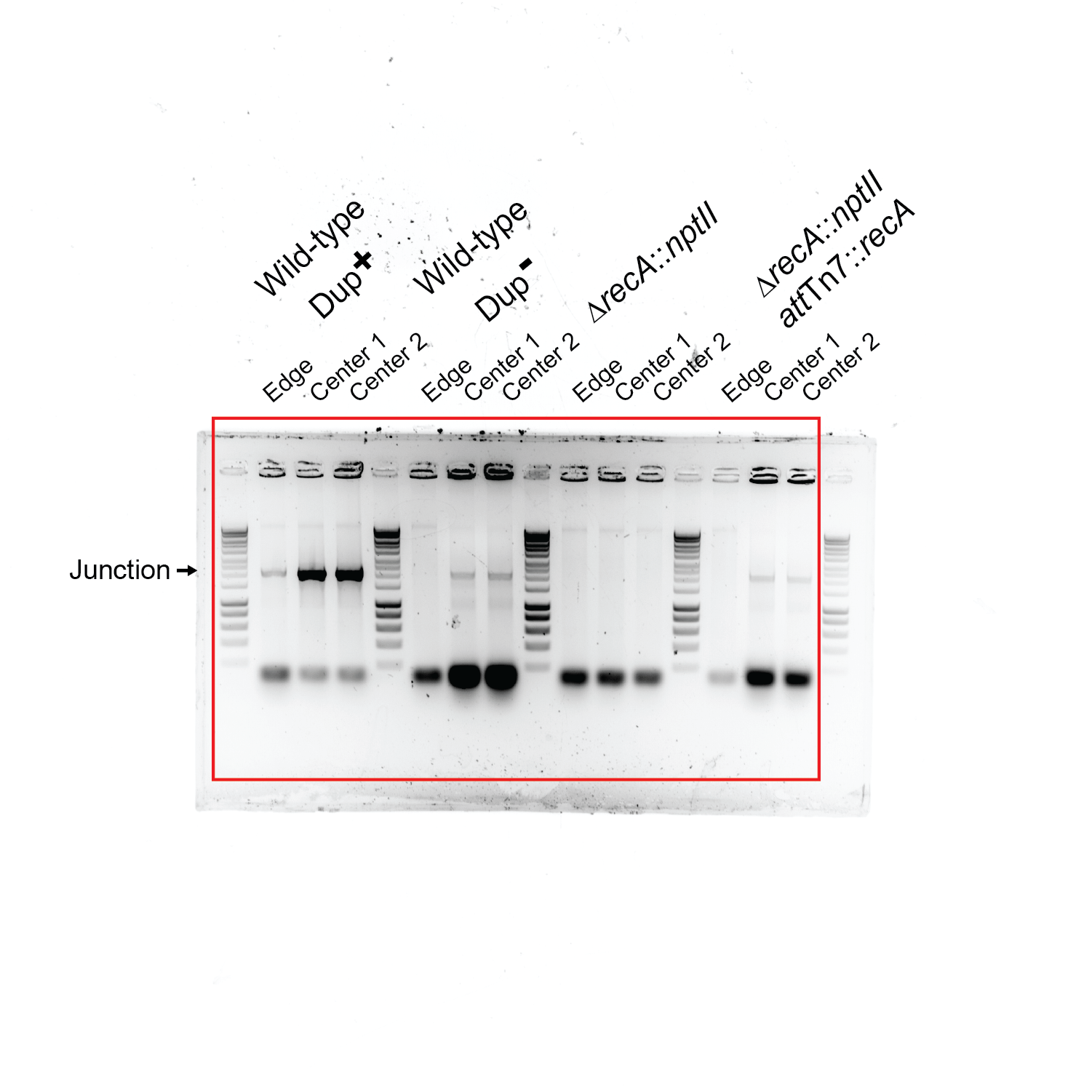

Supplement: Figure 7—source data 1. — The red box indicates the region of the gel used in the final figure. The lanes and identity of the band(s) are indicated. [file elife-84327-fig7-data1.zip › Figure 7 - Source data 1/Figure 7 - Source data 1.png]

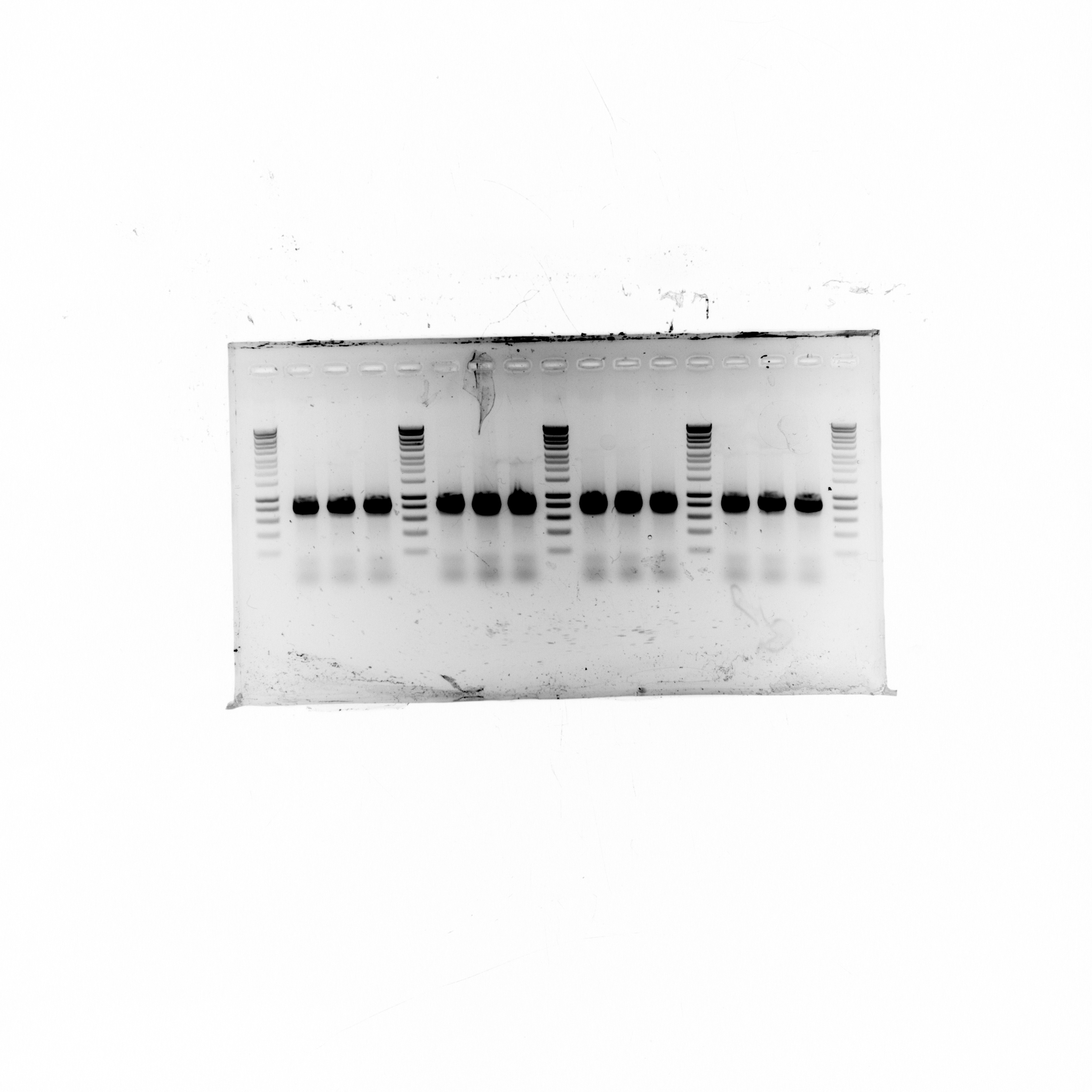

Supplement: Figure 7—source data 2. — The red box indicates the region of the gel used in the final figure. The lanes and identity of the band(s) are indicated. [file elife-84327-fig7-data2.zip › Figure 7 - Source data 2/Figure 7 - Source data 2 unedited.tif]

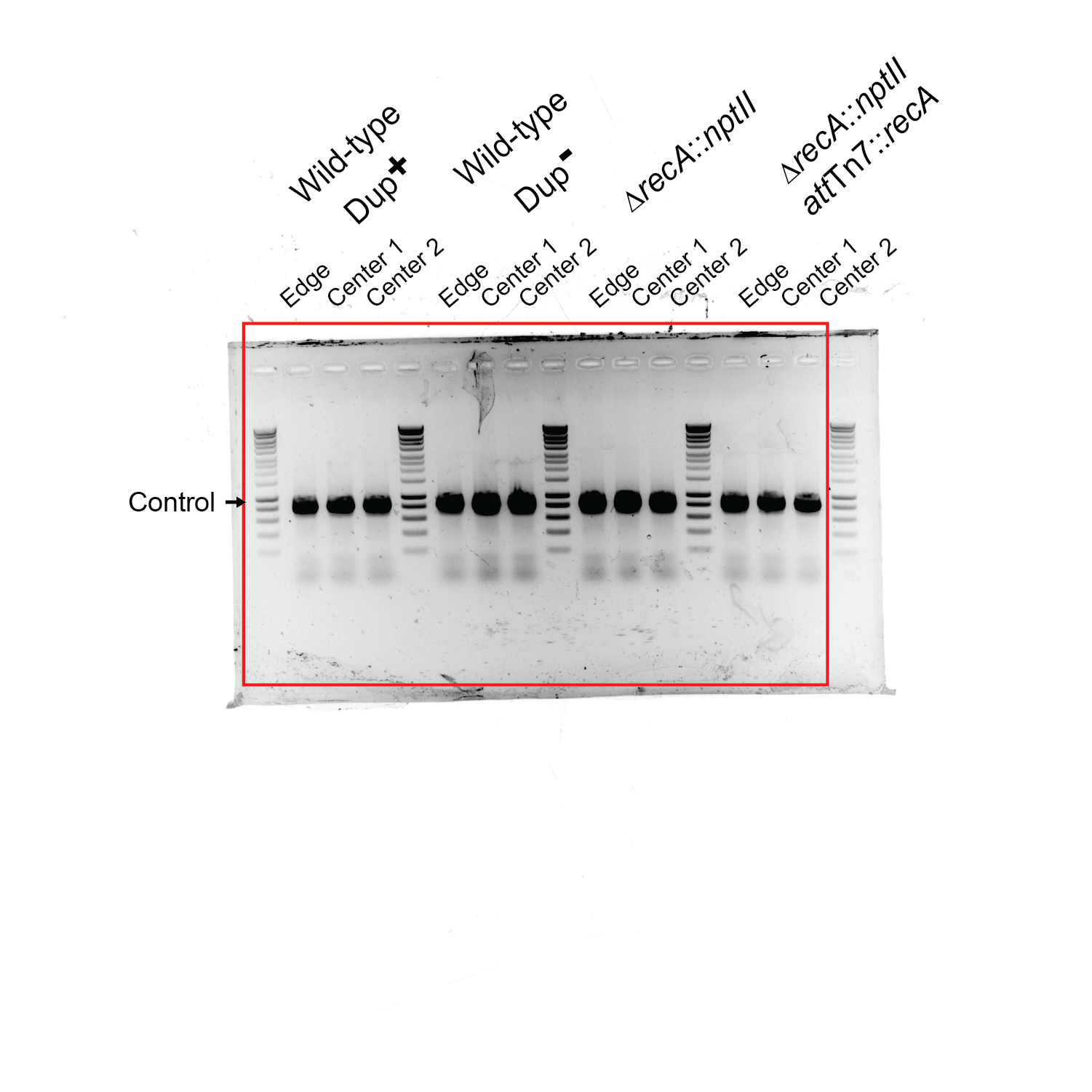

Supplement: Figure 7—source data 2. — The red box indicates the region of the gel used in the final figure. The lanes and identity of the band(s) are indicated. [file elife-84327-fig7-data2.zip › Figure 7 - Source data 2/Figure 7 - Source data 2.png]
